# Whole Exome Sequencing Identifies Frequent Somatic Mutations in Cell-Cell Adhesion Genes in Chinese Patients with Lung Squamous Cell Carcinoma

**DOI:** 10.1038/srep14237

**Published:** 2015-10-27

**Authors:** Chenguang Li, Zhibo Gao, Fei Li, Xiangchun Li, Yihua Sun, Mengyun Wang, Dan Li, Rui Wang, Fuming Li, Rong Fang, Yunjian Pan, Xiaoyang Luo, Jing He, Liangtao Zheng, Jufeng Xia, Lixin Qiu, Jun He, Ting Ye, Ruoxin Zhang, Minghui He, Meiling Zhu, Haichuan Hu, Tingyan Shi, Xiaoyan Zhou, Menghong Sun, Shilin Tian, Yong Zhou, Qiaoxiu Wang, Longyun Chen, Guangliang Yin, Jingya Lu, Renhua Wu, Guangwu Guo, Yingrui Li, Xueda Hu, Lin Li, A Asan, Qin Wang, Ye Yin, Qiang Feng, Bin Wang, Hang Wang, Mingbang Wang, Xiaonan Yang, Xiuqing Zhang, Huanming Yang, Li Jin, Cun-Yu Wang, Hongbin Ji, Haiquan Chen, Jun Wang, Qingyi Wei

**Affiliations:** 1 Department of Thoracic Surgery, Fudan University Shanghai Cancer Center, Shanghai, 200032, China; 2 Department of Oncology, Shanghai Medical College, Fudan University, Shanghai, 200032, China; 3 BGI-Shenzhen, Shenzhen, 518083, China; 4 Cancer Institute, Fudan University Shanghai Cancer Center, Shanghai, 200032, China; 5 State Key Laboratory of Cell Biology, Institute of Biochemistry and Cell Biology, Shanghai Institute for Biological Sciences, Chinese Academy of Science, Shanghai, 200031, China; 6 Department of Medical Oncology, Fudan University Shanghai Cancer Center, Shanghai, 200032, China; 7 Department of Pathology, Fudan University Shanghai Cancer Center, Shanghai, 200032, China; 8 State Key Laboratory of Genetic Engineering and MOE Key Laboratory of Contemporary Anthropology, School of Life Sciences and Institutes of Biomedical Sciences, Fudan University, Shanghai, 200433, China; 9 Lab of Molecular Signaling, Division of Oral Biology and Medicine, School of Dentistry and Jonsson Cancer Center, UCLA, Los Angeles, California, 90095, USA; 10 Duke Cancer Institute, Duke University Medical Center, and Department of Medicine, Duke University School of Medicine, Durham, North Carolina, 27710, USA; 11 The Novo Nordisk Foundation Center for Basic Metabolic Research, University of Copenhagen, 2200, Denmark; 12 Department of Biology, University of Copenhagen, Copenhagen, 2200, Denmark; 13 BGI-Shanghai, Eastern CHINA, BGI-Shenzhen, Shanghai, 201100, China; 14 Department of Medicine and Therapeutics, State Key Laboratory of Digestive. Disease, Li Ka Shing Institute of Health Sciences, The Chinese University of Hong Kong, Hong Kong

## Abstract

Lung squamous cell carcinoma (SQCC) accounts for about 30% of all lung cancer cases. Understanding of mutational landscape for this subtype of lung cancer in Chinese patients is currently limited. We performed whole exome sequencing in samples from 100 patients with lung SQCCs to search for somatic mutations and the subsequent target capture sequencing in another 98 samples for validation. We identified 20 significantly mutated genes, including *TP53*, *CDH10*, *NFE2L2* and *PTEN*. Pathways with frequently mutated genes included those of cell-cell adhesion/Wnt/Hippo in 76%, oxidative stress response in 21%, and phosphatidylinositol-3-OH kinase in 36% of the tested tumor samples. Mutations of Chromatin regulatory factor genes were identified at a lower frequency. In functional assays, we observed that knockdown of *CDH10* promoted cell proliferation, soft-agar colony formation, cell migration and cell invasion, and overexpression of *CDH10* inhibited cell proliferation. This mutational landscape of lung SQCC in Chinese patients improves our current understanding of lung carcinogenesis, early diagnosis and personalized therapy.

Lung cancer remains the leading cause of cancer-related death worldwide^1^. Adenocarcinoma and squamous cell carcinoma are two major subtypes of non-small cell lung cancer (NSCLC), which accounts for nearly 85% of all lung cancer cases, with a 5-year survival rate as low as 16%^2^. Novel drugs targeting the ‘driver’ mutations (e.g., *EGFR* tyrosine kinase mutations and *ALK* fusions) have led to prolonged survival and better quality of life for patients with lung adenocarcinoma^3^, in contrast to limited benefits for lung squamous cell carcinoma (SQCC), because current knowledge on lung SQCC tumorigenesis is still limited, especially in Chinese patients.

Previous studies have identified several mutated genes in lung SQCCs, such as *TP53, EPHA2, NFE2L2, AKT1, LKB1, PTEN* and *ERBB2*^4^,^5^,^6^,^7^,^8^,^9^, as well as copy number gains of *SOX2*, *FGFR1* and *PDGFRA*^10^,^11^,^12^, which have served as candidate driver genes for targeted therapy. Recent next-generation sequencing studies have showed a comprehensive genomic landscape of lung SQCCs in both Caucasian and Korean patients^13^,^14^. Frequently altered genes identified in these studies included *TP53*, *NFE2L2*, *KEAP1*, *CUL3*, and *CSMD3*. Nevertheless, the mutational landscape shown in these studies doesn’t explain all lung SQCC cases, and functional study on key mutated genes and pathways in lung SQCC is absent. Furthermore, no studies of genomic landscape of lung SQCCs in Chinese patients have reported to date. Therefore, additional evidence from genomic level sequencing and functional studies of lung SQCCs is needed.

In the present study, we searched for somatic mutations comprehensively in 198 Chinese lung SQCC patients, of which 100 cases undergone initial whole exome sequencing as a discovery cohort and an additional 98 cases for target capture sequencing as a prevalence cohort of independent samples. We aimed to uncover the genomic landscape and to find the key mutated genes for lung SQCCs in Chinese patients.

## Results

### Samples and clinical data

We initially screened by the whole exome sequencing of genomic DNA from tumors and paired normal lung tissues of 100 patients with untreated stage I-III lung SQCC as a discovery cohort (Supplementary Table 1) and followed by the target capture sequencing of an additional 98 paired samples as a prevalence cohort (Supplementary Table 2). In the target capture sequencing, we focused on significantly mutated and potentially important functional genes observed in the discovery cohort. Overall, 87.9% (174/198) of the patients had a history of smoking, similar to those found in studies of Caucasian population^13^. We isolated and measured genomic DNA by the standard quality-control methods.

### Gene mutational status in lung SQCCs

We generated reads with the Illumina HiSeq 2000 platform (Illumina Inc, San Diego, CA) and aligned them against the reference human genome (hg18). We found that 98.71% of the target regions were represented by at least one uniquely mapped read in both paired normal and tumor tissues, with a corresponding average sequencing depth of 48.2-fold and 49.4-fold in the exome sequencing, respectively. In addition, over 87.3% and 87.0% of the target regions for normal and tumor tissues, respectively, were covered sufficiently for confident variant calling (defined as ≥10X). Added by target capture sequencing, the sequencing depth in the target regions reached 100X. The mean and median mutation rate was 4.40 and 4.33 mutations per Mb (range, 0.04 to 11.64), respectively.

We used several rigorous bioinformatics approaches to identify reliable somatic mutations. To eliminate germline variants reported previously, we filtered our sequence data through the database of dbSNP132 and the 1000 Genome Project. In the discovery cohort, we identified a total of 11,979 somatic substitutions, including 2,287 synonymous, 8,004 missense, 634 nonsense and 18 stop-loss mutations, 505 splice-site changes and 4,971 mutations in UTR, intronic and intergenic regions, in addition to 134 small coding insertions or deletions (indels) (data file 1–2). The mutation spectrum of substitutions was dominated by C:G > A:T tranversions (31%), followed by C:G >T:A transitions (26%) that are most likely to be smoking specific^15^. Then, we performed validation for 621 somatic substitutions and all of the 134 coding indels. We confirmed 560 (90.2%) somatic substitutions and 79 (79%, out of 100 samples successfully sequenced) somatic indels by Sanger sequencing (data file 3–4). To further validate these findings, we performed the target capture sequencing in another independent 98 pairs of samples from patients with lung SQCCs as a prevalence cohort with a 100X sequencing depth, targeting on significantly mutated and potentially important genes detected in the discovery cohort (data file 5–7).

**Figure 1:**
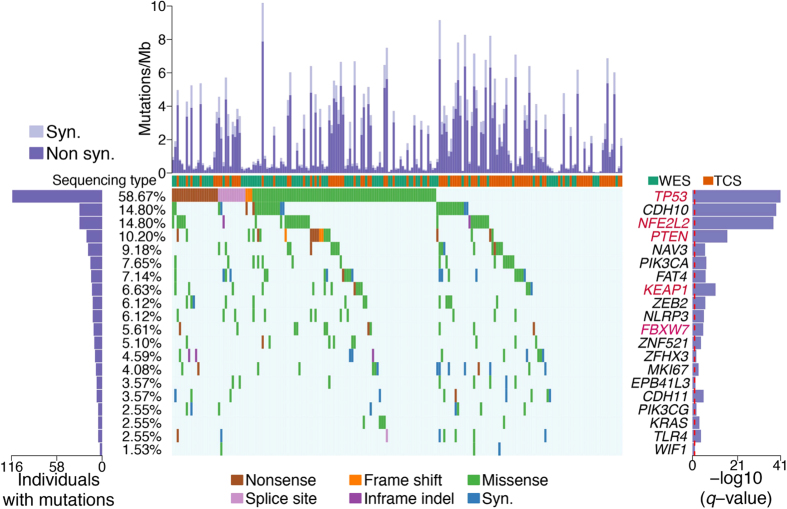
Significantly mutated genes in lung SQCC. Significantly mutated genes (Qvalue < 0.1) identified by exome and target capture sequencing of 198 lung SQCCs are listed vertically by Q-value. The percentage of individuals with mutations of each gene is shown on the left. Samples displayed as columns and the overall number of mutations is plotted at the top. SMGs identified by both algorithms are marked with red font color. Samples screened by whole exome sequencing are marked with green font color, samples screened by target capture sequencing are marked with orange font color. Syn., synonymous; WES, whole exome sequencing; TCS, target capture sequencing.

**Figure 2:**
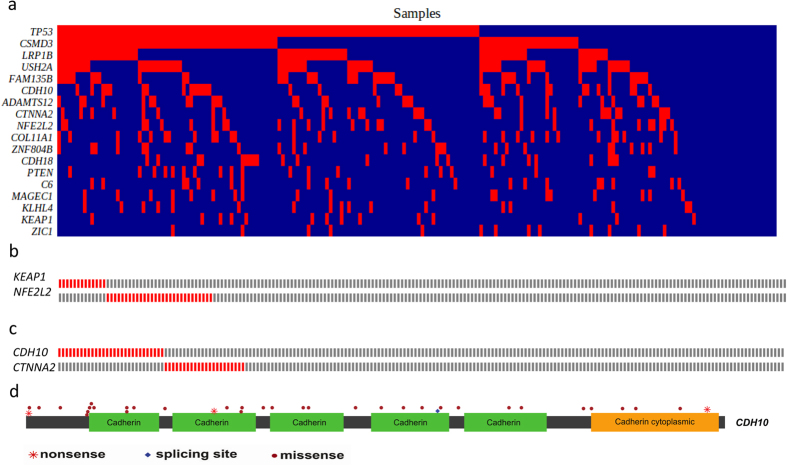
Mutually exclusive/concurrent mutated genes and somatic mutations in *CDH10* in lung SQCCs. (**a**) Concurrent and mutually exclusive mutations observed in frequently mutated genes in lung SQCCs. For each gene (row), tumors (columns) with or without mutations are labeled in red or blue, respectively. (**b–c**) Totally mutual exclusive (no mutations from the two genes in one sample) in several pairs of gene, such as *KEAP1* and *NFE2L2*, *CDH10* and *CTNNA2*, are shown. (**d**) Somatic mutations in *CDH10*. The types and relative positions of somatic mutations are shown in the transcripts of *CDH10* using the following symbols: red stars, nonsense mutations; bullets, missense mutations; blue diamonds, mutations at splice sites. Domains and motifs in each encoded protein product are also indicated.

We screened for the significantly mutated genes (SMGs) by MutSigCV^16^. As a result, we identified 6 significantly mutated genes including *TP53*, *NFE2L2*, *PTEN*, *KEAP1*, *FBXW7* and *TMPRSS13* with a false discovery rate (FDR) *Q*-value < 0.1. To identify key genes that would be missed by MutSigCV, we employed an algorithm present below by focusing only on cancer related genes (see **Methods**). Finally, we identified 20 SMGs from the exome and target capture sequencing data of 198 lung SQCC samples (Fig. 1). Seven of the 20 genes have been previously reported to be mutated in lung SQCCs, which are *TP53*^4^,^17^,^18^, *KEAP1*^13^,^14^,^19^, *NEF2L2*^6^,^13^,^20^, *PTEN*^14^, *KRAS*^13^, *PIK3CA*^13^,^14^ and *CDH10*^21^. The tumor-normal lung mRNA expression levels of these 20 genes were queried from the TCGA Pan-Cancer dataset^22^ (Supplementary Fig. 1). Of these significantly mutated genes, *CDH10* attracted us because of the high *Q*-scores and potential functions. Mutations in the pair of *CDH10* and *CTNNA2* (*P* = 0.002) or *KEAP1* and *NFE2L2* (*P* = 0.026) were mutually exclusive, indicating a functional interaction that is involved in lung tumorigenesis (Fig. 2a–c). Mutations in *CDH10* were largely distributed throughout the entire gene allele, without recurrent mutation sites or specific localized areas (Fig. 2d).

### CDH10 potentially plays a tumor-suppressor role in lung SQCC

Several lines of evidence suggest that *CDH10* potentially play a tumor-suppressor role. Of the 41 mutations identified in the 198 samples analyzed (20 in the discovery cohort and 21 in the prevalence cohort), four were predicted to be inactivating mutations (three nonsense and one splice-site mutation) (Fig. 2d). Seven of 33 samples with *CDH10* mutations had two or three independent mutations, presumably inactivating two alleles. In the sample 1019LC, for example, we identified two nonsynonymous mutations (*i.e. NM_006727:c.C2059T:p.R687W* and *NM_006727:c.T1883C:p.V628A*), and the first was predicted to affect a cadherin domain. Intriguingly, there were 38 reads/read-pairs that spanned across both mutations, of which 7 supported the first mutation (i.e. *NM_006727:c.C2059T:p.R687W*), while 11 supported the second (i.e. *NM_006727:c.T1883C:p.V628A*), but none of these 7 or 11 reads/read-pairs supported both (Supplementary Fig. 2). Therefore, it is likely that these two mutations may target each of the two alleles separately, leading to the bi-allelic inactivation of *CDH10*, which meets the requirement of the “two-hits” model of a tumor-suppressor gene. *CDH10* has been reported to be mainly expressed in human brain and prostate^23^, we also compared *CDH10* expression levels among lung, brain and prostate tissues (Supplementary Fig. 3–4). Western-blot analysis and qualitative PCR confirmed that that *CDH10* is expressed in the lung at a lower level than in brain and prostate, which is consistent with previously published studies^16^. In the functional assays using two lung cancer cell lines with high *CDH10* expression (NCI-H522 and NCI-H1373) (Supplementary Fig. 5 a,b), *CDH10* silencing using different shRNAs consistently resulted in a significant increase in cell proliferation (Supplementary Fig. 6 a,f) and soft-agar colony formation (Supplementary Fig. 6 g,h). We further found that *CDH10* silencing also promoted cell migration (Supplementary Fig. 6 i,j) and cell invasion in matrigel culture (Supplementary Fig. 6 k,l). Knockdown of *CDH10* in another cell line (NCI-1437) also significantly increased cell proliferation (Supplementary Fig. 5 c,d). Overexpression of *CDH10* significantly inhibited cell proliferation in wild-type mouse embryonic fibroblasts (MEFs) and mouse lung cancer cell line L793 (Supplementary Fig. 7). Collectively, these functional studies further demonstrated that *CDH10* might serve as a tumor suppressor in lung carcinogenesis.

### Frequently mutated pathways in lung SQCC

**Figure 3:**
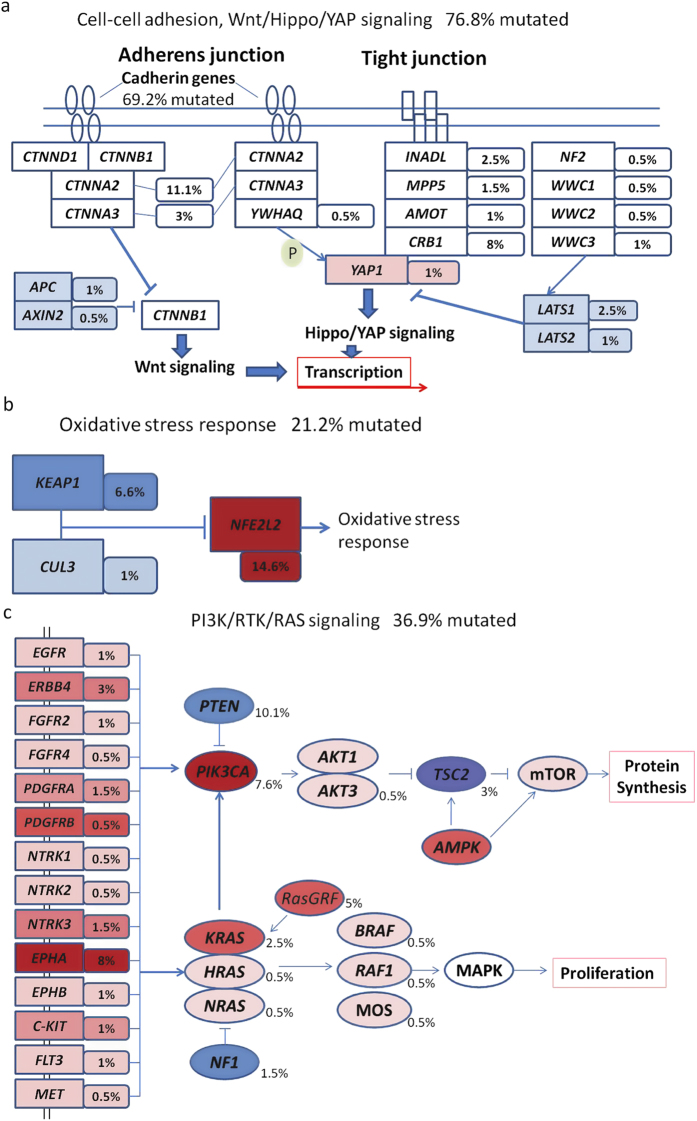
Significantly mutated pathways in lung SQCC. Somatic mutations in lung SQCC frequently occurred in genes of the (**a**) Cell-Cell adhesion/Wnt/Hippo/YAP, (**b**) Oxidative stress response, (**c**) PI3K/RTK/RAS signaling pathways. Oncogenes are indicated in pink to red and tumor suppressor proteins are shown in light to dark blue. The darkness of the colors is positively correlated to the percentage of tumors with genetic alterations. The frequency of genetic alterations for each of these pathway members in 198 tumors is indicated.

Frequent somatic mutations in specific biological pathways indicate their unique roles in tumorigenesis and progression. To identify key mutated pathways of lung SQCCs, we firstly screened mutated genes through published papers on NSCLC mutational spectrum; secondly, frequently and potentially important mutated genes were searched in KEGG and Cell Signaling pathway database manually. In the present study, except for the well-studied *TP53* mutations, other pathways with frequently mutated genes included those of cell-cell adhesion/Wnt/Hippo in 76.8%, oxidative stress response in 21.2%, and phosphatidylinositol-3-OH kinase in 36.9% of the tested tumors. Most remarkably, genes involved in the cell-cell adhesion/Wnt/Hippo signaling pathway were frequently affected by somatic mutations (Fig. 3a). We observed somatic mutations of several cadherin genes, of which *CDH10* (14.6%) was the most frequently mutated, and other mutated cadherin genes included *CDH18* (8%), *FAT4* (7%) and *PCDH15* (8.6%). Overall, 69.2% of samples had non-silent mutations of the cadherin superfamily genes (data file 8). It is known that α-catenin is a binding partner of the cadherins encoded by *CTNNA1*, *CTNNA2* and *CTNNA3*, and the latter two genes were mutated in 11.1% and 3% of the tested samples, respectively. Furthermore, α-catenin links cell-cell junction genes to the Wnt and Hippo/YAP signaling pathway, modulating cell-cell adhesion and contact inhibition. Other mutated genes in this pathway included *CRB1*, *LATS1/2* and *YAP1*, suggesting that alteration of the cell-cell adhesion/Wnt/Hippo signaling pathway may play the vital role in lung SQCC.

The oxidative stress response pathway protects cells against oxidative and xenobiotic damage, and genetic alterations of *NFE2L2* and *KEAP1* have been previously reported in NSCLC^13^,^24^,^25^. In the present study, *NFE2L2* and *KEAP1* were mutually exclusively mutated in 14.6% and 6.6% of the tested samples (*P* = 0.026), respectively, accounting for 21.2% of the 198 lung SQCC samples (Fig. 3b,c). *NFE2L2* encodes a transcriptional factor that induces cytoprotective proteins upon oxidative damage, and the cellular *NFE2L2* level is regulated by *KEAP1*^26^. All of the eight mutations identified in *NFE2L2* in the present study were in exon 2, which is the key region that interacts with *KEAP1*^20^. These findings confirmed previous studies that *NFE2L2* and *KEAP1* were frequently mutated and played important roles in lung SQCCs^13^,^20^. Mutations of genes in the phosphatidylinositol-3-OH kinase pathway have been previously reported in NSCLC, especially in lung adenocarcinoma^13^,^27^. In the present study, 36.9% of samples harbored at least one non-silent mutation in genes of this pathway (Fig. 3c). Although the receptor kinase genes were much less frequently mutated in lung SQCC than in lung adenocarcinoma, *PIK3CA* and *PTEN* were commonly altered in lung SQCCs in the present study.

### Somatic mutations affected chromatin regulatory factors in lung SQCCs

The chromatin regulatory factors referred to those proteins that regulate chromatin structure, thus maintain genome integrity and regulate gene expression^28^. Genetic alteration of these group of genes have been reported in various types of cancer, mainly including renal cell carcinoma^29^, bladder cancer^30^, endometrium^31^, breast cancer^32^ and so on. We annotated the mutated genes in our study to the list of likely driver chromatin regulatory genes reported previously^28^, and found a 27.3% overall mutation rate of these genes, which was also largely mutual exclusive (Supplementary Fig. 8a). Interestingly, we found a trend of complete mutual exclusive correlation between these genes when they were gathered as SWI/SNF and PRC protein complexes (Supplementary Fig. 8b,c), with an overall mutation rate of 13.6% and 7.1%. These above results suggest that chromatin regulatory genes are affected by somatic mutations in lung SQCCs, largely in the form of protein complexes rather than individual genes.

### Clinical-pathological and mutual association analyses of frequently mutated genes

Genes that play key roles in tumor initiation and progression often exhibit strong associations with clinical-pathological characteristics, in a mutually exclusive or concurrent relationship with other mutated genes. Therefore, we performed additional analyses to further uncover potential oncogenes and tumor-suppressor genes in lung SQCCs. Pairs of mutually exclusive genes included *NFE2L2* and *KEAP1* (*P* = 0.026), *CTNNA2* and *CDH10* (*P* = 0.002), *CTNNA2* and *C6* (*P* = 0.023), *ZEB2* and *KIF2B* (*P* = 0.033), *CRB1* and *NFE2L2* (*P* = 0.012) and other pairs of genes (Fig. 2a–c, data file 9).

In the present study, we found that mutations in *CSMD3* were associated with early TNM stage (*P* = 0.001) and significantly less frequent in N2 than N0 and N1 tumors (*P* = 0.007). As a gene with very long coding region, *CSMD3* mutations were frequently identified but not significantly mutated than background mutation. In a previous study, loss of *CSMD3* resulted in increased proliferation of airway epithelial cells^33^. As a potential candidate tumor suppressor, loss of heterozygosity or homozygous deletions may occur in genomic area of *CSMD3*^34^,^35^, however, we did not find loss of copy number in genomic regions of *CSMD3* in tumor samples compared with normal lung samples (data file 10). Based on current evidences, the function of *CSMD3* gene in lung carcinogenesis needs further validation. We also found that mutations in *PTEN* were associated with early TNM stages (*P* = 0.02) and significantly less frequent in N2 tumors (*P* = 0.002) (Supplementary Fig. 9). Finally, we identified a panel of genes whose frequent mutations were detected only in smokers, including *NFE2L2*, *MAGEC1*, *NLRP3* and *FAM5C*. As most lung SQCC cases occur in patients with smoking history, those mutated genes which were only found in smokers in this study need further validation with lager number of patients. Although smokers and non-smokers did share some mutations of the same genes, we did not find any of other frequently mutated genes that were exclusively found in non-smokers.

## Discussion

As described in previously published sequencing studies of lung SQCC of Caucasian patients^13^,^33^, the present study showed similar characteristics of mutations in Chinese patients, such as high mutation rates of *TP53*, *CSMD3* and genes in the oxidative stress response and *PI3K* pathways, but the present study also uncovered some novel genes (such as the cadherin family genes) and pathways (such as the cell-cell adhesion/Wnt/Hippo pathway), indicating that there are commonly altered functions in cell-cell adhesion, contact inhibition, oxidative stress response and apoptotic signaling in lung SQCC tumorigenesis.

**Figure 4:**
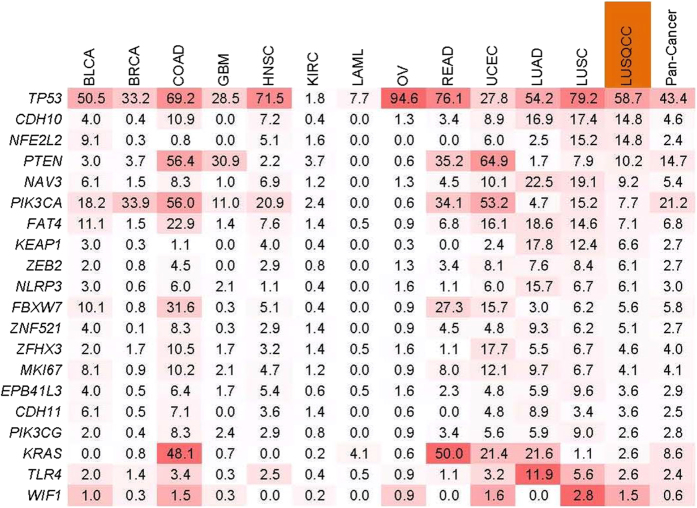
Mutation diversity of SMGs in Chinese lung SQCCs and other cancer types from TCGA data. Percentages of samples mutated in each cancer types and Pan-Cancer are shown, and the highest percentage in each gene is shown in bold. BLCA, bladder urothelial carcinoma; BRCA, breast adenocarcinoma; COAD,READ, colon and rectal carcinoma; GBM, glioblastomamultiforme; HNSC, head and neck squamous cell carcinoma; KIRC, kidney renal clear cell carcinoma; LAML, acute myeloid leukaemia; OV, ovarian serous carcinoma; UCEC, uterine corpus endometrial carcinoma; LUAD, lung adenocarcinoma; LUSC, lung squamous cell carcinoma (TCGA study); LUSQCC, lung squamous cell carcinoma (the present study).

We screened for the mutation frequency of 20 SMGs across TCGA data, which included both 11 other cancer types and lung squamous cell carcinoma (Fig. 4). Both TCGA and our study on lung SQCC showed high mutation frequencies of *TP53*, *NFE2L2* and *PTEN*, indicating similar molecular events in squamous carcinogenesis. *CDH10* was found to be frequently mutated in both lung squamous cell carcinoma and adenocarcinoma than other cancer types, which further indicated the importance of *CDH10* mutations in NSCLC development. Compared with the 178 and 104 lung SQCCs samples from Caucasian patients^13^ and Korean patients^14^, we found similar main SMGs including *TP53*, *KEAP1*, *NFE2L2* and *PTEN* (Supplementary Fig. 10) in Chinese patients. Interestingly, some of those frequently mutated genes identified in the TCGA lung SQCC study were not found in the present study. For example, inactivation of *CDKN2A* caused by somatic mutations was seldom observed in the present and Korean lung SQCC studies. It is possible that other mechanisms, such as methylation and homozygous deletion, may contribute to the loss of *CDKN2A* functions in Asian lung SQCC. The rare *NOTCH1* truncating mutations observed in the present study indicates that squamous differentiation genes may not play key roles in lung SQCC of Chinese patients, different from those in SQCCs of the lung and head and neck reported for Caucasian patients^13^,^36^. *MLL2* was identified as a SMG with a mutation rate as high as 20% in Caucasian and Korean patients. However, *MLL2* mutations were not found in the present study. Other frequently mutated genes found in the Korean study like *CHD7, NF1* and *NOTCH* were much rarer in our study. On the other hand, frequently mutated cell-cell adhesion genes, which gone through target-capture sequencing in our study, were not shown in the Korean study. Our study indicated that cell-cell adhesion gene on the whole are frequently mutated and disordered in lung SQCCs of Chinese patients. Those above differences in significantly and frequently mutated genes among different cohort of patients offer additional evidences of lung SQCC tumorigenesis, which is worth of further investigation.

We also compiled the so-called cancer druggable genes according to DrugBank, NCI, PharmGKB, as well as curated from published papers, which led to 389 drugs with diverse targets, and we selected potentially therapeutic targets through the exome data from the discovery cohort of 100 lung SQCCs. Somatic mutations that had medium or high effects as determined by mutation accessor were calculated, if they could be targeted by one of these 389 drugs. Finally, we identified 63 potential druggable cases, indicating that 63% of lung SQCC patients could potentially receive target therapies (data file 11).

The cadherin-catenin complex is the major player involved in the formation of cell-cell adherens junctions and important for maintaining cell polarity and tissue integrity, which potentially limits cell movement and proliferation^37^. The cadherin genes are characterized by multiple repeats of the cadherin motif in their extracellular domains that are involved in the Ca^2+^ binding, whereas their cytoplasmic domains significantly diverge among these members. Growing lines of evidence suggest that cadherins may play critical roles in control of the proliferative or oncogenic signaling, including the Wnt/β-catenin, Ras/MAPK and Hippo/YAP pathways (Fig. 3a, Supplementary Fig. 11)^37^,^38^. For example, α-catenin has two major subtypes: αE-catenin (encoded by *CTNNA1*) and αN-catenin (encoded by *CTNNA2*). α-catenin maintains structure integrity of the epithelium through interacting with cadherin-α-catenin complexes and cytoskeleton. While *CTNNA2* was less studied, over-expression of αN-catenin, like αE-catenin, in the PC-9 lung carcinoma cell line was able to restore cell-cell adhesion and epithelial morphology^39^. Recently, αE-catenin was found to be the key molecule in the Hippo signaling pathway, controlling cell proliferation by interacting with YAP1^38^,^40^,^41^. Abnormal activation of YAP due to loss of αE-catenin led to squamous cell carcinoma-like tumors in the mouse skin^38^. The integrity of the cadherin-catenin complex is negatively regulated by phosphorylation of β-catenin, receptor tyrosine kinases (RTKs) and cytoplasmic tyrosine kinases, which leads to dissociation of the cadherin-catenin complex. The Wnt signaling acts as a positive regulator of β-catenin by inhibiting β-catenin degradation. Further, cadherin may acts as a negative regulator of β-catenin by binding to β-catenin at the cell surface and sequesters it from the nucleus. Taken together, our results suggest that among other unknown mechanisms, dysregulation of the cadherin-catenin complex caused by somatic mutations may promote lung SQCC formation by modulating multiple signaling pathways.

In summary, we identified both the well-known and unreported somatic mutations in lung SQCCs by using the exome sequencing of 100 followed by target capture sequencing of 98 paired samples of tumors and their adjacent normal tissues in Chinese patients. We uncovered a high somatic mutation frequency of the cell-cell adhesion/Wnt/Hippo pathway genes in lung SQCC and demonstrated that *CDH10* exerted tumor suppressor role. Our results illustrated the landscape of the mutation spectrum of lung SQCC in Chinese patients and provided valuable candidate genes for further investigations.

## Methods

### Tissue collection and DNA isolation

A total of 198 lung SQCC tissue samples and paired normal lung tissue samples were collected between Oct, 2007 and Dec, 2011 at Fudan University Shanghai Cancer Center, Shanghai, China. Clinical data were collected in the perioperative period (Supplementary Table 1–2). After initial 100 paired tumor-normal lung samples were screened by the whole exome sequencing as a discovery cohort, and an independent group of additional 98 paired tumor-normal lung samples underwent the target-capture sequencing as a prevalence cohort to validate initial important findings. All patients provided a written consent form before recruitment into the study. This study was approved by the Ethics Review Board of Fudan University Shanghai Cancer Center and the methods were carried out in accordance with the approved guidelines. Fresh surgical samples were snap-frozen and stored in liquid nitrogen upon resection until use. Each tumor sample was determined as a primary lung SQCC by pathologists, with a minimum of 70% of tumor cellularity, and all patients did not receive neoadjuvant chemotherapy. DNA of tumors and paired normal lung tissue was isolated by using standard protocols of QIAamp DNA Mini Kit (Qiagen, Hilden, Germany). A total amount of 5 μg DNA of each sample was used for the exon capture. The quality of genomic DNA was examined by both spectrophotometer (Nanodrop, Thermo Fisher Scientific Inc.) and gel-electrophotometric method.

### Illumina-based whole-exome sequencing

Genomic DNA from tumors and normal tissues was fragmented and hybridized to commercially available capture arrays for enrichment. The whole exome capture procedure was performed with Agilent’s SureSelect Human All Exon Kit protocol (Agilent SureSelect Human All Exon Kit 38MB). Resulting DNA libraries with an insert size of 200 bp on average were sequenced using the 90-bp paired-end technology on Illumina Hiseq 2000. Real-time image analysis and base calling were performed by Hiseq Control Software version 1.1.37 and Real Time Analysis version 1.7.45 using standard parameters, respectively.

### Reads mapping and mutations detection

After removing reads containing sequencing adapters and low-quality reads with more than five unknown bases, the high quality reads were aligned to the NCBI human reference genome (hg18) using BWA^42^ (v0.5.9) with the default parameters. Picard^43^ (v1.54) was employed to mark duplicates and followed by Genome Analysis Toolkit^44^ (v1.0.6076) (GATK IndelRealigner) to improve alignment accuracy.

Somatic SNVs were detected by VarScan2.2.5^45^ (samtools (v0.1.18) mpileup –Q 0 && VarScan2.2.5 somatic --min-coverage 10 --min-coverage-normal 10 --min-coverage-tumor 10 --min-var-freq 0.1 --min-avg-qual 0). Somatic indels were predicted with GATK SomaticIndelDetector with default parameters. All high confident mutations were obtained using in house pipeline coupled with visual inspection, then annotated with ANNOVAR^46^ (released time 2011-10-02).

### Validation of somatic mutations by Sanger sequencing

To validate somatic SNVs and indels with Sanger sequencing, PCR primers designed for the putative somatic variants were used to amplify the DNA from the tumors first. If the mutations were confirmed in the tumors, the same primer pairs were used to amplify the DNA of normal lung tissues from the same subjects to determine somatic status of the mutations. Detailed information of validation is shown in Data file 3–4.

### Illumina-based target-capture sequencing

After the whole exome sequencing of 100 pairs of samples as a discovery cohort, we identified significantly mutated genes by NS:S statistics as described previously^27^,^47^. We also observed a number of potentially important genes that were frequently mutated in lung SQCCs, such as the cadherin superfamily genes. To validate these important findings in a large number of independent samples as a prevalence cohort, we ordered the Target Capture Array from Angilent corporation, targeting 193 genes (Data file 7) identified from the discovery cohort.

### Identification of significantly mutated genes

We first applied MutSigCV^16^ to identify significantly mutated genes (SMGs) in the exome and target capture sequencing cohorts of 198 SQCCs, which produced 6 SMGs (i.e. *TP53, NFE2L2, PTEN, KEAP1, FBXW7* and *TMPRSS13*) with *q*-value < 0.1, this probably due to relatively small number of mutations and large number of genes (18,862 in total) being used in MutSigCV, leading to many multiple hypothesis test corrections. To identify key genes that would be missed by MutSigCV, we employed an algorithm present below by focusing only on cancer related genes. We began by obtaining background mutation rates for each category (i.e. ***C*** = {*CpG- > T, *Cp(A/C/T)- > *T, A-* > *G, transver, and null* + *indel*}) from MutSigCV^1^. For the ease of description, the background mutation rates for these five categories are denoted as ***r*** = {r_*CpG->__*T*_, r_*Cp(A/C/T)->*T*_*, r*_*A-*>*G*_*, r*_*transver*_*, r*_*null+indel*_}, the number of bases for each category in gene denoted as ***n***_*g*_ = {*n*_g.*CpG->*T*_*, n*_g.*Cp(A/C/T)->*T*_*, n*_*g.A-*>*G*_*, n*_*g.transver*_*, n*_*g.null+indel*_}. In this case, the expected number of somatic mutations in gene *g* was calculated as:


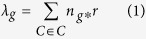


The *p*-value of observing *X*_*g*_ ≥ *K* somatic mutations in gene *g* given the expected mutation count *λ*_*g*_ is:


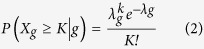


The Benjamini Hochberg method of false discovery rate control (FDR-BH) was used to convert *p*-values into *q*-values. This algorithm produced 20 SMGs.

### Functional study of *CDH10* in lung cancer cell lines

#### Cell culture

Human lung cancer cell lines NCI-H522 and NCI-H1373 were purchased from ATCC (American Tissue Culture Collection, the Global Bioresource Center) and used for functional studies within six months after thaw from liquid nitrogen tank. All cells were cultured in DMEM medium (Hyclone) supplemented with 8% fetal bovine serum (FBS) (Biochrom AG, Germany) and antibiotics (100 U/ml streptomycin and 100 μg/ml penicillin) (Invitrogen, USA). The human cell line HEK-293T cells were maintained in DMEM (Hyclone) supplemented with 8% fetal bovine serum (FBS) and antibiotics. L793 cell line is a mouse cell line derived from lung SQCC tumor of *Kras*^*G12D*^*/Lkb1*^*L/L*^ *(Kras/Lkb1)* mice^48^.

#### Plasmids and lentivirus package

The human *CDH10* sequence was amplified from NCI-H522 cDNA using primers:

5′-CGGAATTCGCCACCATGGACTACAAGGACGACGATGACAAGACAATACATCAATTTTTGCTACTGTTTC-3′ (forward, EcoRI-flag-F)

5′-CGGGATCCTTAAGAGTCTTTGTCACTTTCCCCAC-3′ (reverse, BamHI-R)

Amplicon was inserted into pCDH-Puro.

The shRNAs towards human CDH10 were cloned into pLKO.1 (Addgene). The target

sequences are:

shCDH10 -1:

5′-CGGCGAGATATTATTCCAGAA-3′

shCDH10 -2:

5′-CGGTACTGATATGTTTGACAT-3′

#### RT-PCR and real-time PCR

We extracted the total RNA by using the Trizol reagent (Invitrogen) according to the manufacturer’s instructions and reverse-transcribed into cDNA (Ferments).

The cDNAs were then used for either regular PCR or real-time PCR on a 7500 Fast Real-Time PCR System (Applied Biosystems) using the SYBR-Green Master PCR mix (Invitrogen). GAPDH served as the internal control.

Primers used were:

Human CDH10: 5′-CGCCAGAGTCATTTACAGCA-3′ (forward) and

5′-ATGTCTTTGGCCTGGATGAC-3′ (reverse);

Human GAPDH: 5′-AGGTGAAGGTCGGAGTCAAC-3′ (forward) and

5′-AGTTGAGGTCAATGAAGGGG-3′ (reverse).

#### The MTT assay

Cells were plated in 96-well plates (Nest) at a density of 2500 cells per well and cell viabilities were detected at indicated time points. At different time points (Day1–Day5), 20 μl of MTT (Sigma) solution (5 mg/ml) was added into each well. Cells were incubated at 37 °C for 4 h. Then the medium was removed and 100 μl DMSO was added to each well to solubilize the formazan crystals. Absorbance was measured at dual wavelength modes (595 nm and 650 nm) using a Microplate Reader (Thermo, MULTISKAN MK3, USA). The values were normalized by defining the value of day1 as 1.

#### Soft agar assay

Cells were suspended on a top layer of DMEM containing 8% FBS and 0.4% agar (Gibco/Invitrogen) in 6-well plates in triplicate and plated on a bottom layer of DMEM containing 8% FBS and 1% base agar. Colonies were photographed and stained with 0.005% crystal violet for 1 hr after 2-week culture and then colonies were counted and analyzed by Quantity One.

#### Wound healing assay

Cells at 100% confluence were starved in the serum-free medium for over 12 hours dependent on different cell lines. Cells were then lightly and quickly scratched with a pipette tip. The cell migration kinetics were closely monitored and photographed using a light microscope (Leica).

#### Three dimensional cell culture

Cells were seeded in the medium contain 2% matrigel (BD) on the top of another layer of solidified matrigel. Cells were then cultured at 37 °C incubators and monitored for the appearing morphologic changes in 1~2 weeks. Photos were taken using a light microscope (Leica).

#### Immunohistochemistry staining and immunoblotting analyses

Histology immunohistochemical and immunoblotting analyses were performed according to standard protocols, as previously described^49^. *CDH10* antibodies used in these assays were bought from Abgent (Catalog: AP1482c) and LifeSpan (Catalog: LS-C138484) company.

## Additional Information

**Accession code**: All sequencing data from this study are deposited in NCBI Sequence Read Archive, under the accession number SRA104856.

**How to cite this article**: Li, C. *et al.* Whole Exome Sequencing Identifies Frequent Somatic Mutations in Cell-Cell Adhesion Genes in Chinese Patients with Lung Squamous Cell Carcinoma. *Sci. Rep.* **5**, 14237; doi: 10.1038/srep14237 (2015).

## Supplementary Material

Supplementary Information

Supplementary Data file 1

Supplementary Data file 2

Supplementary Data file 3

Supplementary Data file 4

Supplementary Data file 5

Supplementary Data file 6

Supplementary Data file 7

Supplementary Data file 8

Supplementary Data file 9

Supplementary Data file 10

Supplementary Data file 11
