# Supplementary material for: Whole Exome Sequencing Identifies Frequent Somatic Mutations in Cell-Cell Adhesion Genes in Chinese Patients with Lung Squamous Cell Carcinoma
Source: Sci Rep. 2015 Oct 27;5:14237. doi: 10.1038/srep14237 (PMC4621504; doi:10.1038/srep14237)
Supplement: Supplementary Information [file srep14237-s1.pdf]

Whole Exome Sequencing Identifies Frequent Somatic Mutations in Cell-Cell Adhesion Genes in Chinese Patients with Lung Squamous Cell Carcinoma

Chenguang Li, Zhibo Gao, Fei Li, Xiangchun Li, Yihua Sun, Mengyun Wang, Dan Li, Rui Wang, Fuming Li, Rong Fang, Yunjian Pan, Xiaoyang Luo, Jing He, Liangtao Zheng, Jufeng Xia, Lixin Qiu, Jun He, Ting Ye, Ruoxin Zhang, Minghui He, Meiling Zhu, Haichuan Hu, Tingyan Shi, Xiaoyan Zhou, Menghong Sun, Shilin Tian, Yong Zhou, Qiaoxiu Wang, Longyun Chen, Guangliang Yin, Jingya Lu, Renhua Wu, Guangwu Guo, Yingrui Li, Xueda Hu, Lin Li, Asan, Qin Wang, Ye Yin, Qiang Feng, Bin Wang, Hang Wang, Mingbang Wang, Xiaonan Yang, Xiuqing Zhang, Huanming Yang, Li Jin, Cun-Yu Wang, Hongbin Ji, Haiquan Chen, Jun Wang, Qingyi Wei

Supplementary Figure 1

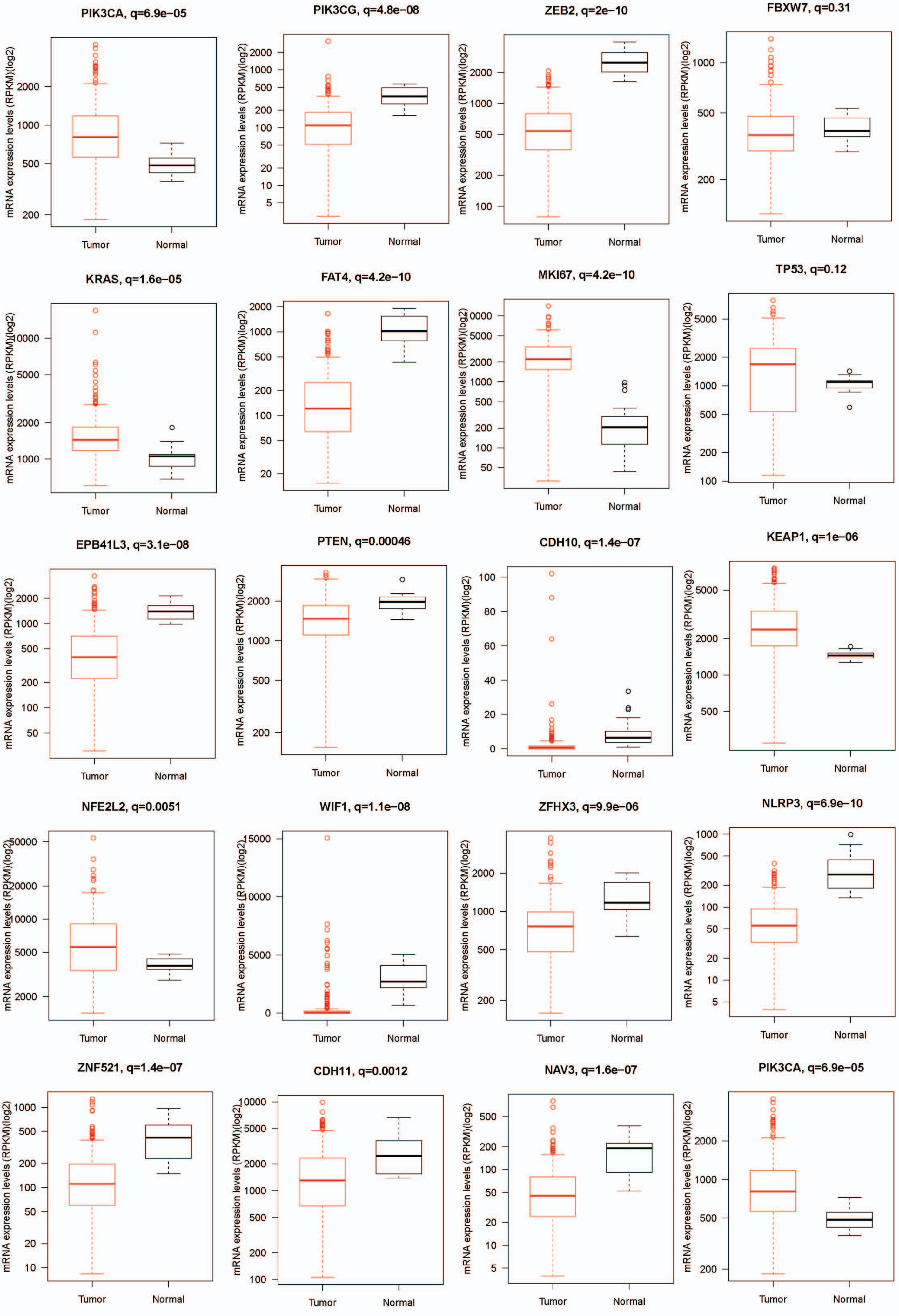

Supplementary Figure 2

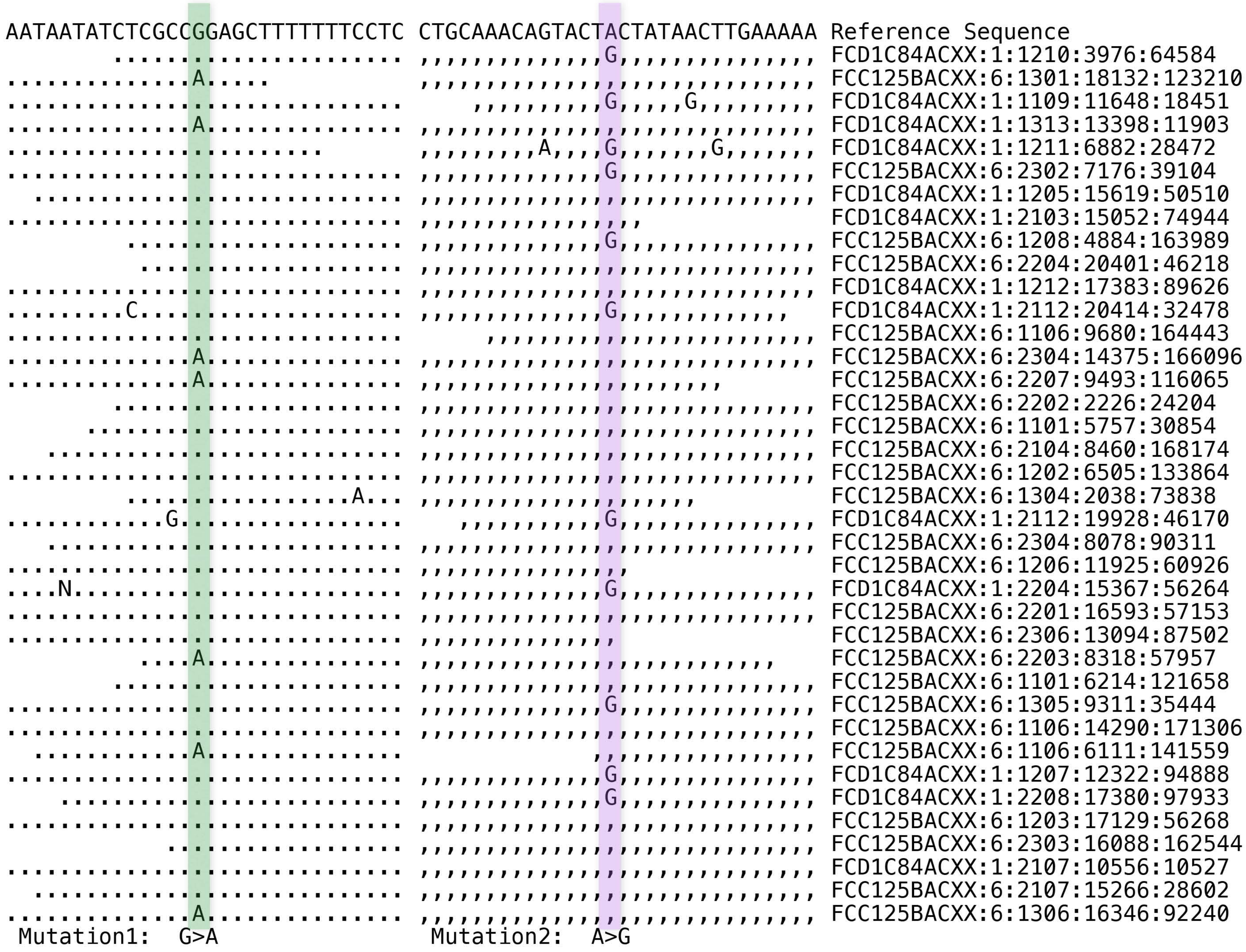

Supplementary Figure 3

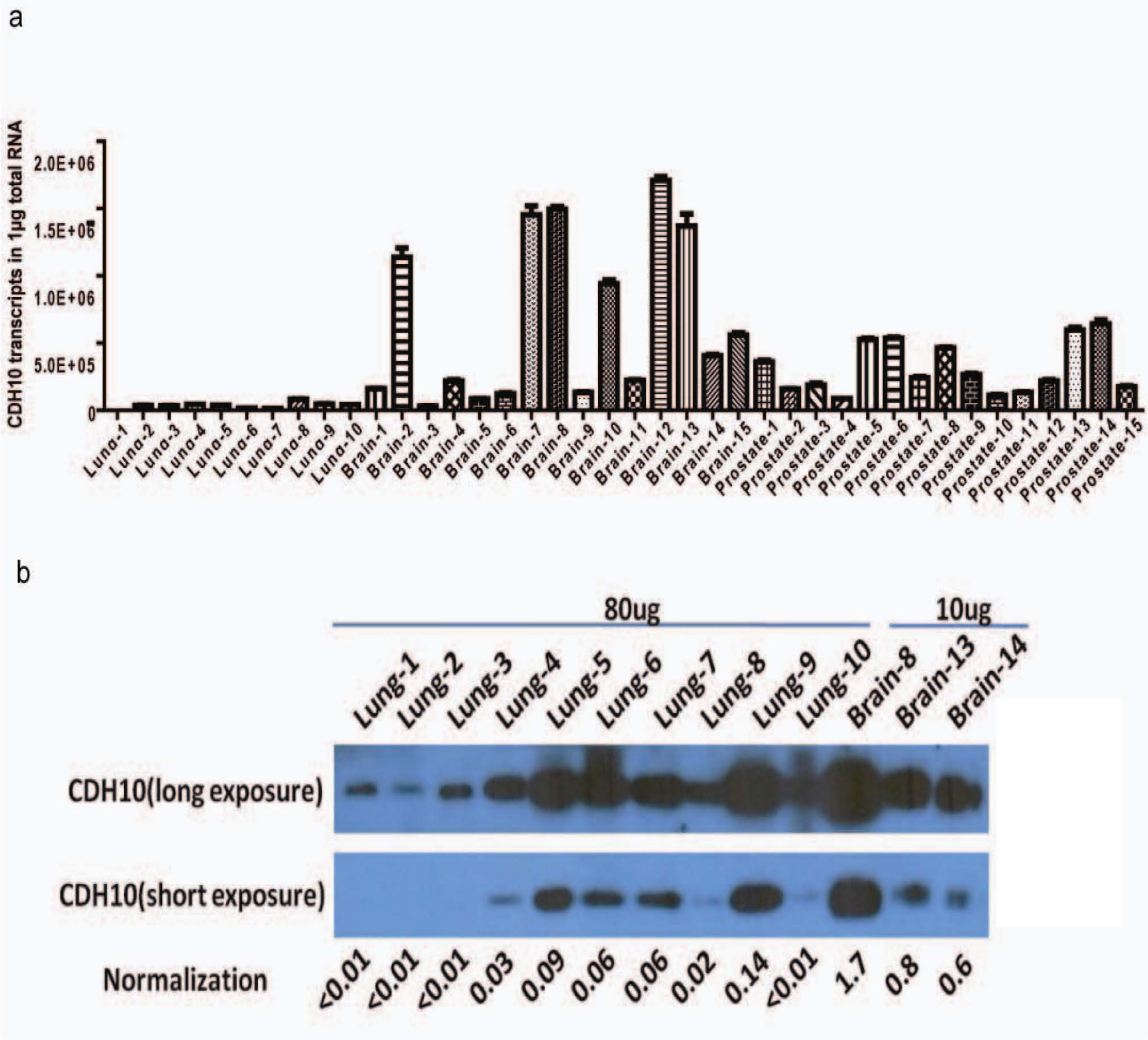

Supplementary Figure 4

a

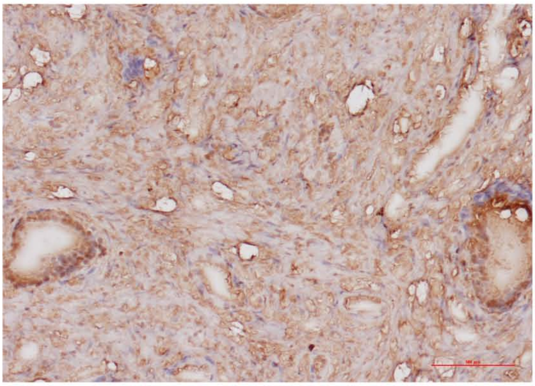

200X

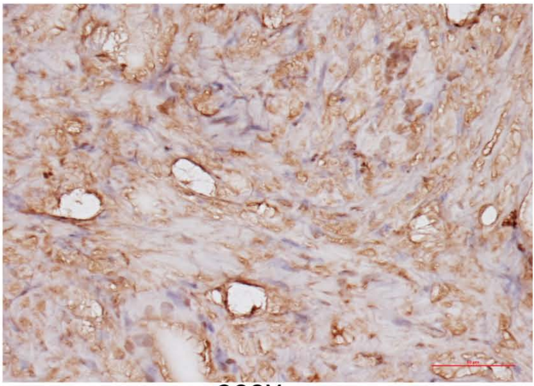

200X

b

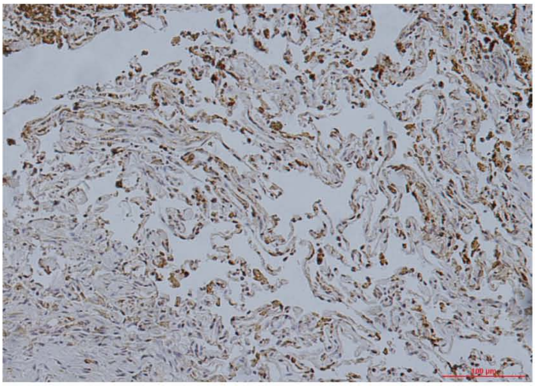

100X

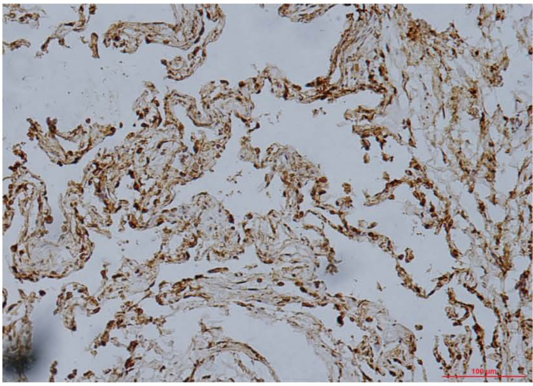

100X

c

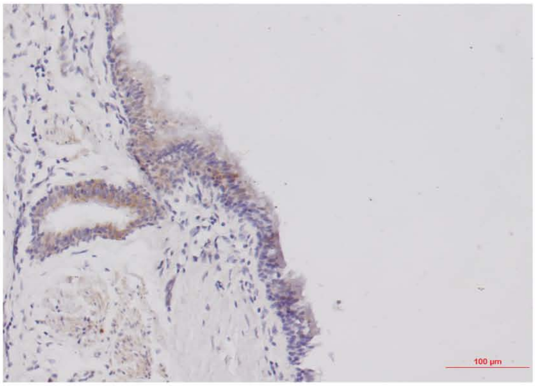

200X

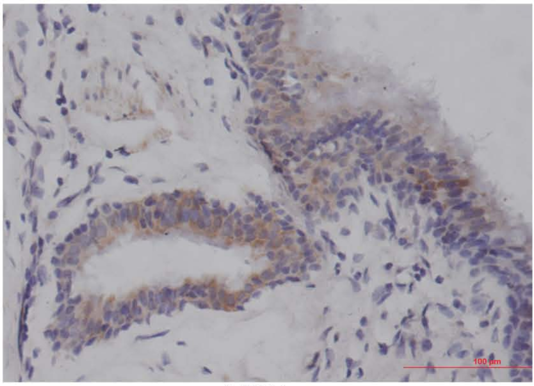

400X

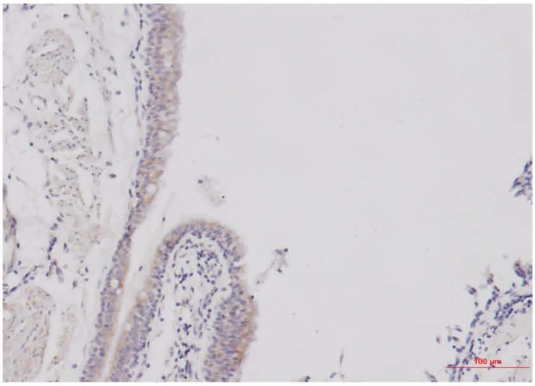

200X

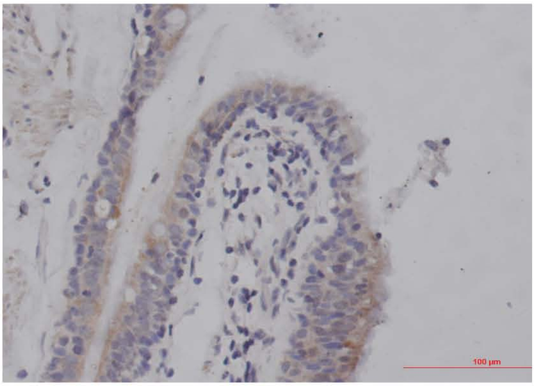

400X

Supplementary Figure 5

a

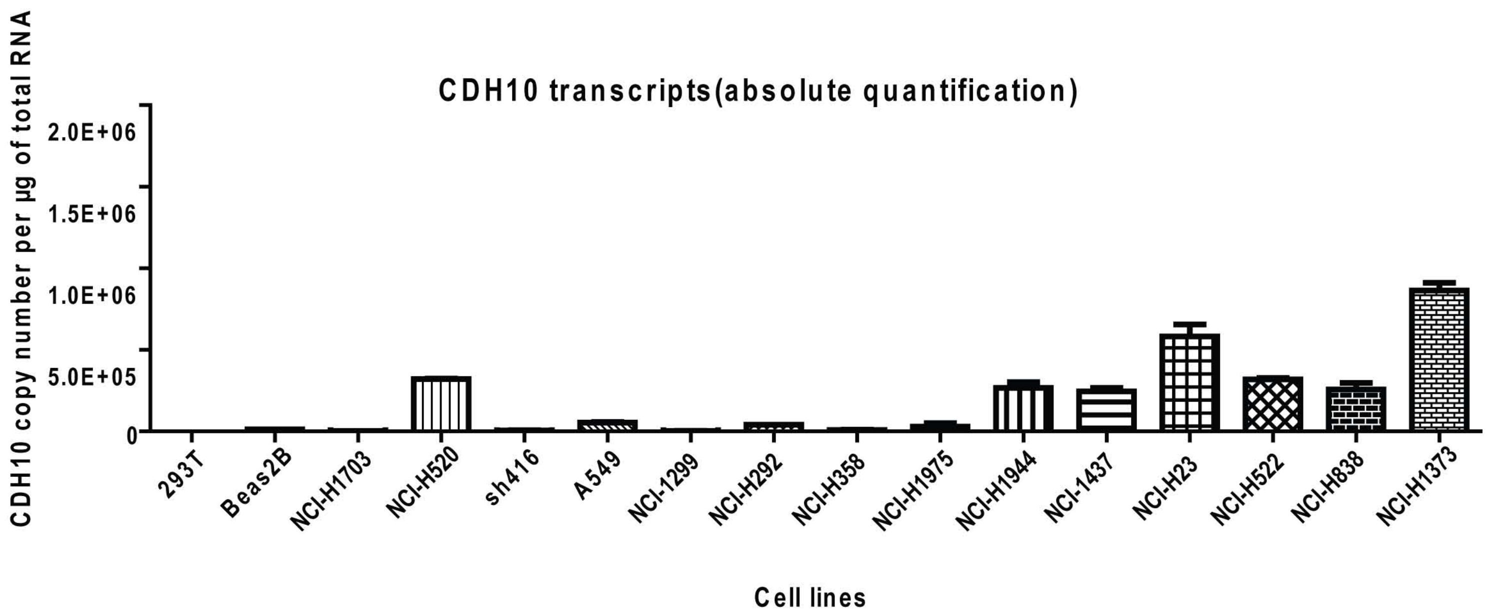

b

NCI-H522						NCI-H1373					
Scramble		shCDH10-1		shCDH10-2		Scramble		shCDH10-1		shCDH10-2	
GAPDH	CDH10	GAPDH	CDH10	GAPDH	CDH10	GAPDH	CDH10	GAPDH	CDH10	GAPDH	CDH10
16.596	24.061	15.899	25.801	16.067	25.506	16.188	23.371	15.76	25.189	15.937	24.888
16.458	23.977	15.854	25.784	16.091	25.542	16.264	23.412	16.044	25.289	15.583	24.714
	24.206		25.697		25.69		23.229		25.372		24.907

c

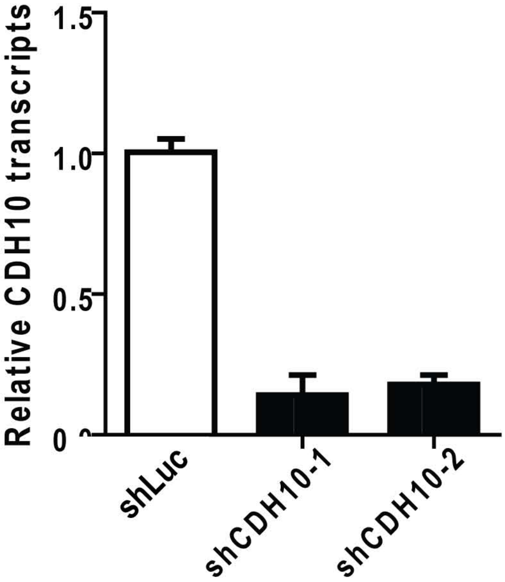

d

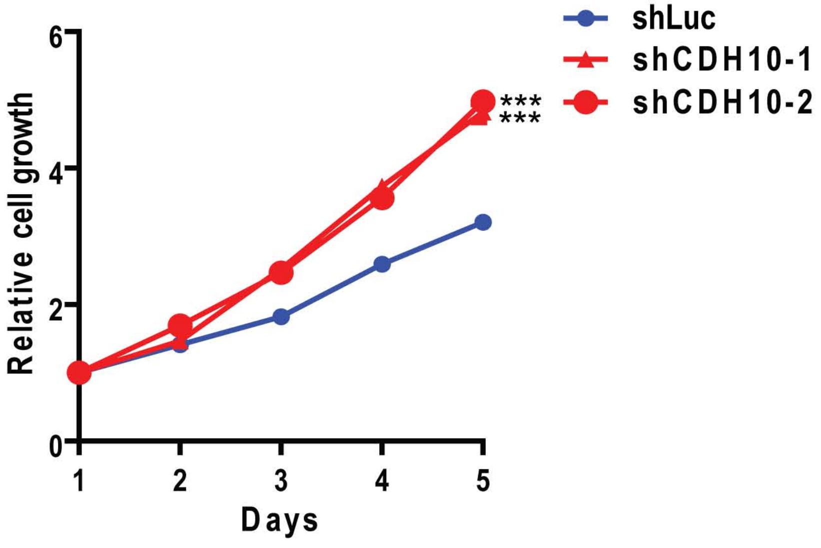

Supplementary Figure 6

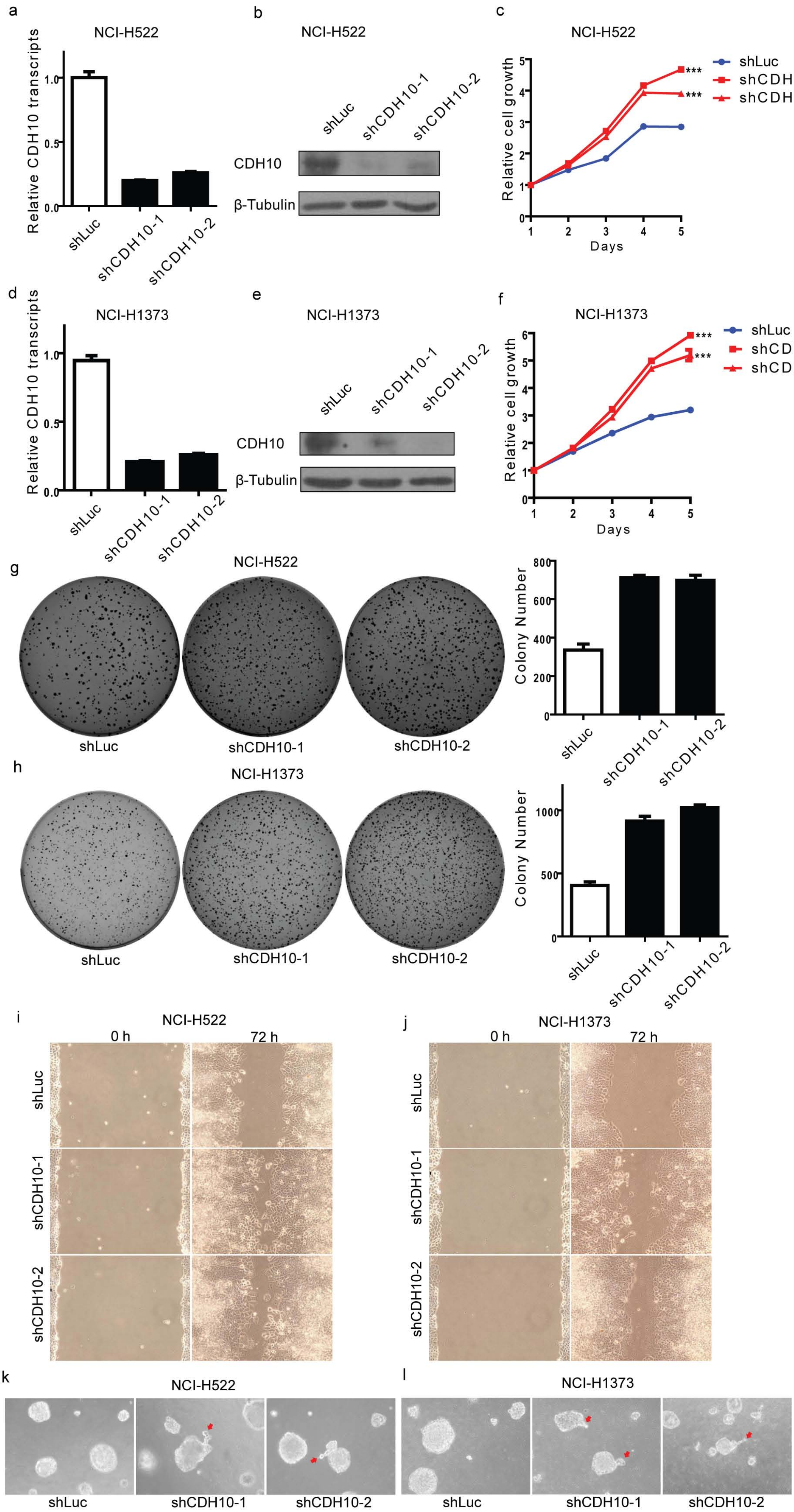

Supplementary Figure 7

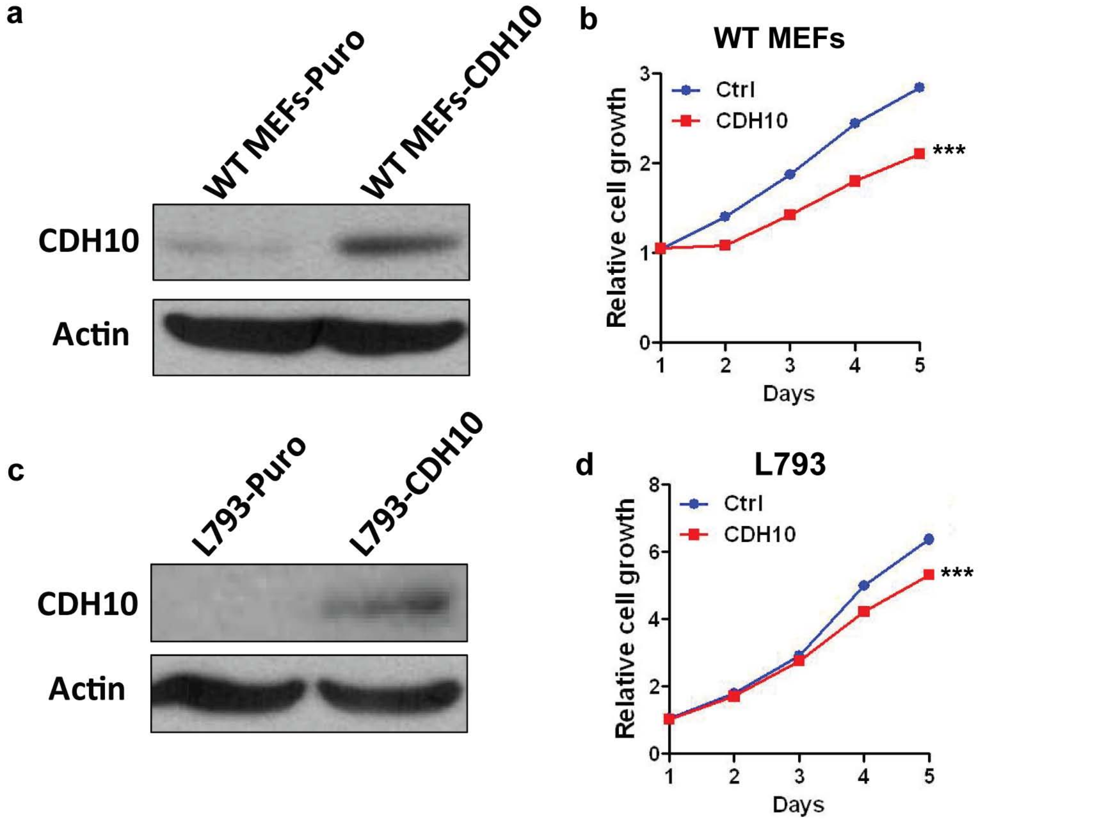

Supplementary Figure 8

a Likely driver chromatin regulatory factor genes, overall mutation frequency 27.3%

b SWI/SNF, overall mutation frequency 13.6%

c PRC, overall mutation frequency 7.1%

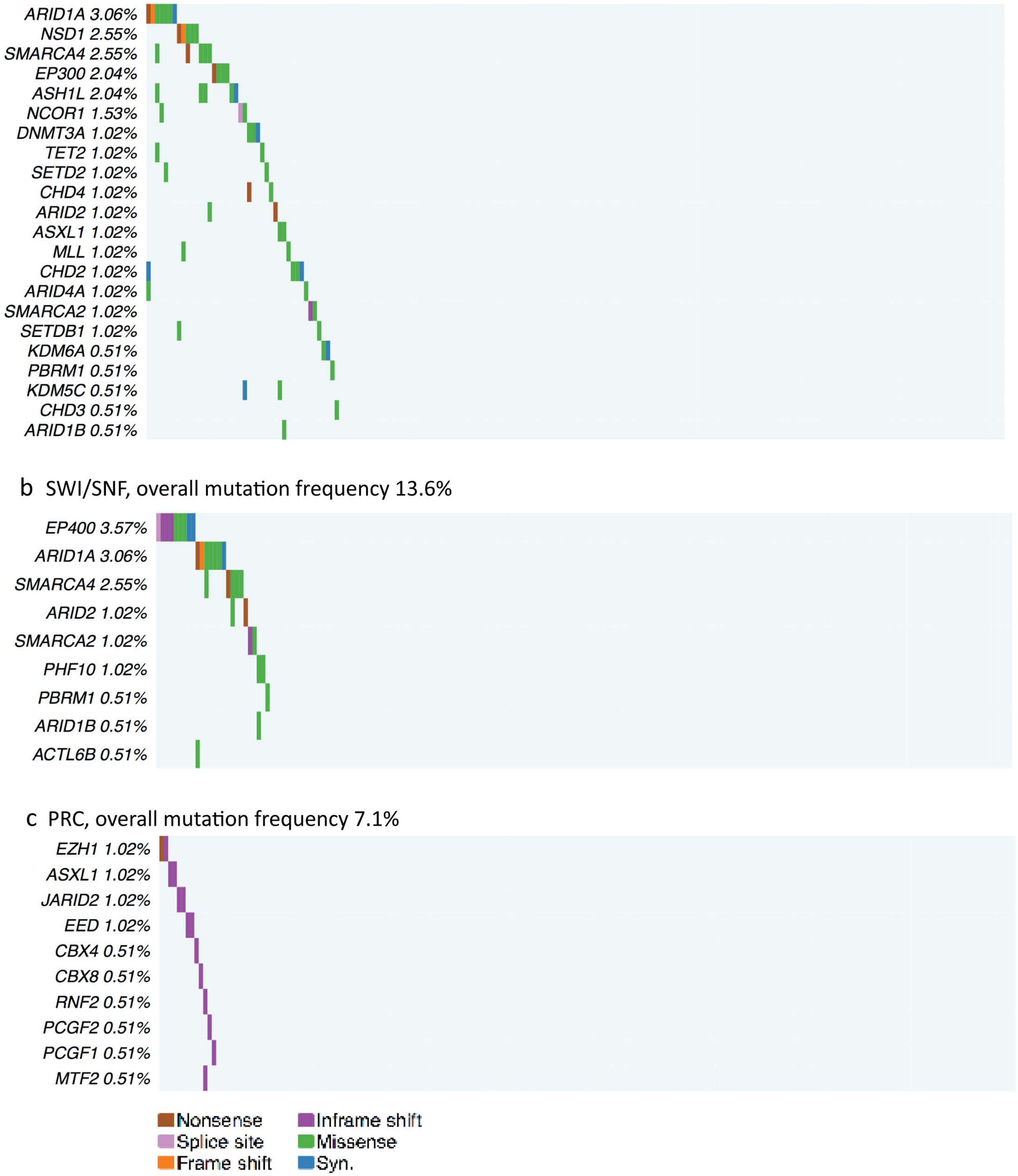

Supplementary Figure 9

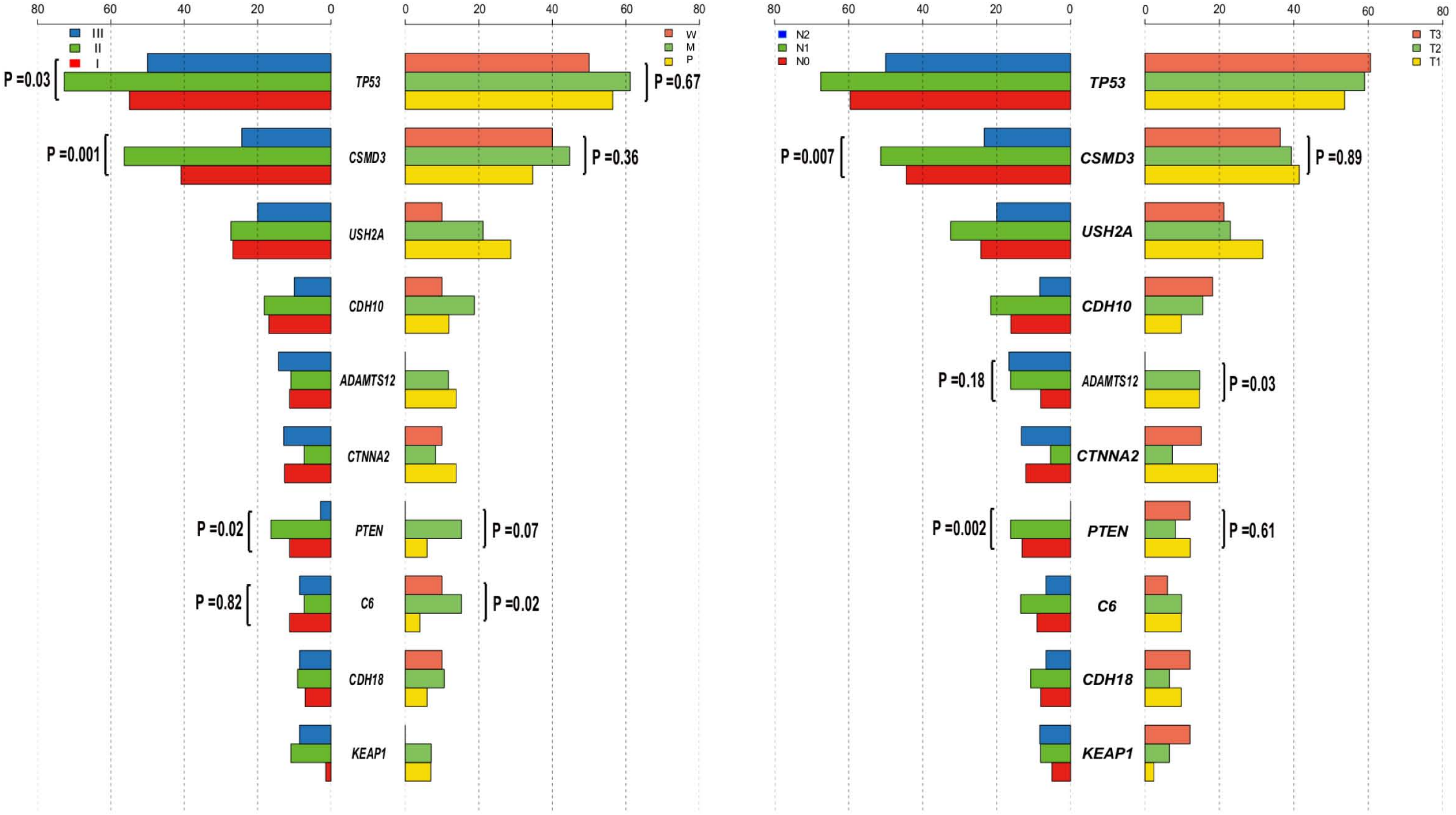

Supplementary Figure 10

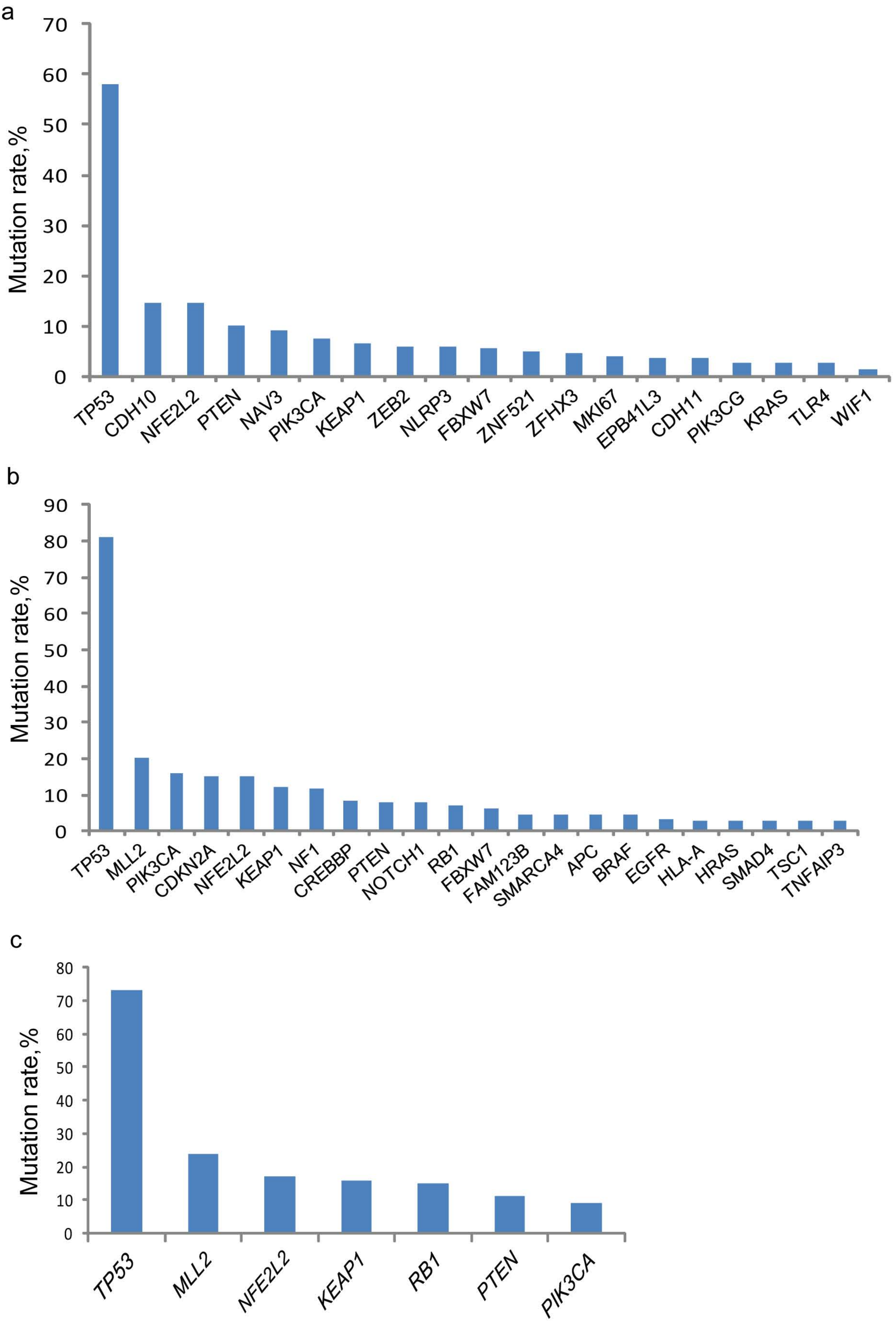

Supplementary figure 11

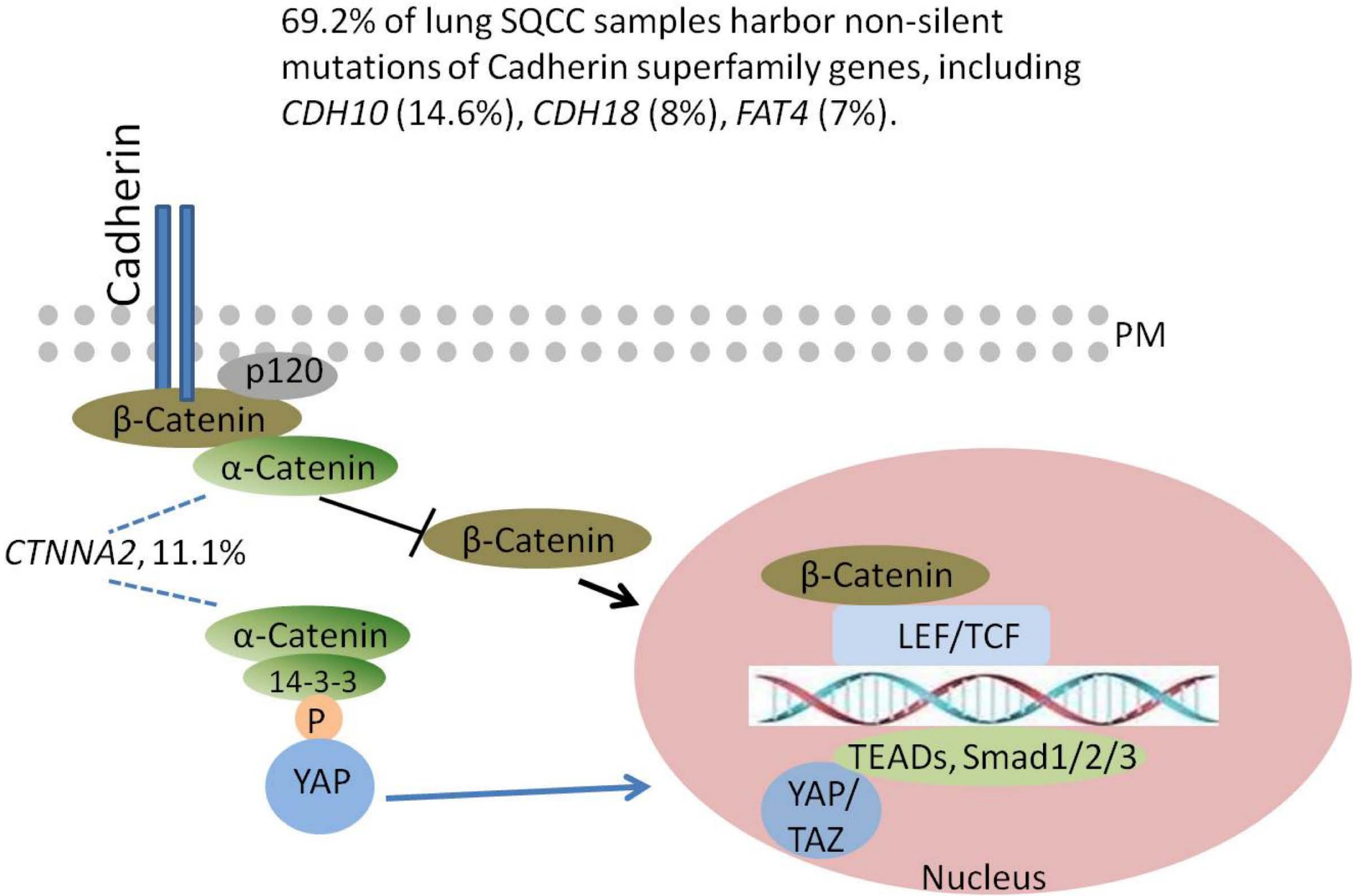

Supplementary Figure Legends

Supplementary Figure 1. mRNA expression level of 20 SMGs in TCGA LUSC cohort.

The tumor-normal lung mRNA expression level of 20 SMGs identified in our study were queried from the TCGA LUSC cohort.

Supplementary Figure 2. Bi-allelic inactivation analysis of CDH10 in sample 1019LC.

Bi-allelic inactivating mutations in CDH10 (extracted by samtools tview), 2 SNVs were encompassed green and violet rectangles, black dot and comma represent nucleotide from forward and reverse strands, respectively.

Supplementary Figure 3. CDH10 expression level in human tissues. (a) The absolute transcript values of CDH10 in 1   g RNA of normal human lung, brain and prostate samples were assessed by quantitative PCR. The CDH10 plasmid (PCDH-puro-CDH10) was used to draw the standard curve for CDH10 absolute transcript measurement. (b) The CDH10 expression was assessed by Western-blot in protein lysate from normal human lung and brain tissues. To make comparison between lung and brain tissues, 80 and 10 μg of normal lung and brain tissue lysates were loaded, respectively. Average level of brain samples were normalized as 1.

Supplementary Figure 4. CDH10 immunohistochemistry staining of prostate (a), pulmonary alveoli (b) and airway epithelial (c), the scale bar was 100μm.

Supplementary Figure 5. CDH10 expression level in cell lines, knockdown efficiency of shCDH10 in cell line NCI-H522 and NCI-H1373 and MTT assay result of NCI-1437. (a) The absolute transcript values of CDH10 in 1 g RNA of each cell line were assessed by quantitative PCR. The CDH10 plasmid (PCDH-puro-CDH10) was used to draw the standard curve for CDH10 absolute transcript measurement. (b) Raw data of quantitative PCR analysis of CDH10 expression in cell lines with CDH10 knockdown. Two cell lines with highest CDH10 expression were chosen for shCDH10 expression. ΔCt were shown. Each experiment was repeated at least three times. (c) CDH10 knockdown efficiency was assessed in NCI-1437 cells through the real time PCR. (d) The MTT assay was performed in NCI-1437 cells with or without CDH10 knockdown, and CDH10 knockdown promoted cell proliferation. *** P<0.001.

Supplementary Figure 6. CDH10 knockdown significantly promotes lung cancer cell proliferation, anchorage-independent cell growth, cell migration and invasion. (a) CDH10 knockdown efficiency was assessed in NCI-H522 cells through the real time PCR. (b) Knockdown of CDH10 in NCI-H522 detected by Western-blot. (c)The MTT assay was performed in NCI-H522 cells with or without CDH10 knockdown, and CDH10 knockdown promoted cell proliferation. *** P<0.001. (d) CDH10 knockdown efficiency was assessed in NCI-H1373 cells through the real time PCR. (e) Knockdown of CDH10 in NCI-H1373 cells detected by Western-blot. (f) The MTT assay was performed in NCI-H1373 cells with or without CDH10 knockdown, and CDH10 knockdown promoted cell proliferation. *** P<0.001. (g-h) The soft agar assay was performed in NCI-H522 (g) or NCI-H1373 (h) cells with or without CDH10 knockdown, and CDH10 knockdown promoted anchorage-independent cell

growth in both cell lines. (i-j) The wound healing assay was performed in NCI-H522 (i) and NCI-H1373 (j) cell lines, and CDH10 knockdown promoted cell migration in both cell lines. The magnification is 200×. (k-l) Cell invasion assay in matrigel was performed in NCI-H522 (k) or NCI-H1373 (l) cells, and CDH10 knockdown promoted cell invasion in both cell lines. Red arrows indicate the cell protrusion. Each experiment was repeated at least three times. The original magnification is 200X.

Supplementary Figure 7. CDH10 overexpression significantly inhibits cell proliferation. (a). Overexpression of CDH10 in WT MEFs detected by western-blot. (b). Overexpression of CDH10 in WT MEFs significantly inhibited cell proliferation. (c). Overexpression of CDH10 in L793 cell line detected by western-blot. (d). Overexpression of CDH10 in L793 cell line significantly inhibited cell proliferation. Each experiment was repeated at least three times. (WT, wild-type; MEFs, mouse embryonic fibroblasts; ***, P<0.001)

Supplementary Figure 8. Mutations from Chromatin regulatory factor genes identified in lung SQCCs. Mutation and mutually exclusive analysis of likely driver Chromatin regulatory factor genes (**a**), SWI/SNF (**b**) and PRC protein complex genes(**c**).

Supplementary Figure 9. Clinical association of frequently mutated genes in lung SQCCs. Clinical-pathological association of the highlighted genes with mutations observed in lung SQCCs, with frequencies of mutations in the highlighted genes by the TNM stage (tumor, lymph node metastasis and distant metastasis), differentiation (W, well differentiated; M,

moderatly differentiated; P, poorly differentiated), N stage (lymph node metastasis alone) and T stage (tumor stage alone). For each stage or differentiation, the frequency of mutations in a given gene was calculated as the proportion of tumors harboring non-silent mutations in the gene among all tumors of the indicated stage or differentiation. The significance of the correlations between mutations and tumor stage or differentiation was determined by Fisher's exact test, two-sided.

Supplementary Figure 10. Comparision of significantly mutated genes identified in three Lung SQCC studies (Adjusted to same analysis tools). (a) Significantly mutated genes in the present study; (b) The TCGA Lung SQCC study; (c) The Korean Lung SQCC study.

Supplementary Figure 11. Overview of the cadherin-catenin and YAP signaling and its regulation. α-catenin binds to β-catenin and inhibits the signaling through the canonical Wnt pathway. YAP acts downstream of α-catenin to control transcription. In this study, 69.2% of lung SQCCs were found to harbor non-silent mutations in cadherin superfamily genes. CTNNA2 was found to be mutated in 11.1% of lung SQCCs.

Supplementary Table 1. Clinical information of 100 Lung SQCC patients

Sample ID	Age (years)	Gender	Smoking status	Differentiation	TNM*	Stage*
7	58	Male	Smoker	M	T3N2M0	IIIa
11	61	Male	Smoker	M	T2aN0M0	Ⅰb
21	65	Male	Smoker	M	T2aN0M0	Ⅰb
25	50	Male	Smoker	M	T2aN0M0	Ⅰb
27	49	Male	Smoker	P	T2aN2M0	IIIa
30	59	Male	Smoker	P	T2aN0M0	Ⅰb
36	59	Male	Smoker	P	T2aN2M0	IIIa
39	48	Male	Smoker	M	T2aN2M0	IIIa
67	68	Male	Smoker	P	T2aN0M0	Ⅰb
94	57	Male	Smoker	M	T2aN0M0	Ⅰb
96	69	Male	Smoker	M	T1aN0M0	Ⅰa
124	61	Male	Smoker	P	T3N2M0	IIIa
127	53	Male	Smoker	M	T2aN1M0	Ⅱa
130	72	Male	Smoker	M	T2aN0M0	Ⅰb
133	64	Male	Smoker	P	T2bN0M0	Ⅱa
134	71	Male	Smoker	P	T3N2M0	IIIa
137	63	Male	Smoker	M	T1bN0M0	Ⅰa
144	54	Male	Smoker	P	T2bN2M0	IIIa
145	57	Male	Non-smoker	M	T1bN0M0	Ⅰa
159	59	Male	Smoker	M	T3N2M0	IIIa
160	48	Male	Smoker	W	T3N1M0	IIIa
170	61	Male	Smoker	P	T2aN3M0	IIIb
171	79	Male	Smoker	P	T1aN0M0	Ⅰa
185	61	Male	Smoker	P	T1bN0M0	Ⅰa
188	50	Female	Non-smoker	P	T2bN2M0	IIIa
192	48	Male	Smoker	P	T2bN0M0	Ⅱa
196	57	Male	Smoker	M	T2aN2M0	IIIa
207	61	Male	Smoker	M	T2bN0M0	Ⅱa
209	62	Male	Smoker	P	T2aN0M0	Ⅰb
214	78	Male	Smoker	M	T1bN0M0	Ⅰa
225	63	Male	Smoker	P	T1aN0M0	Ⅰa
234	55	Male	Smoker	M	T1aN0M0	Ⅰa
257	60	Male	Smoker	M	T3N0M0	Ⅱb
281	65	Male	Smoker	M	T1bN2M0	IIIa
292	54	Male	Smoker	M	T2aN0M0	Ⅰb
298	55	Female	Non-smoker	M	T2aN2M0	IIIa
299	50	Male	Smoker	M	T2aN1M0	Ⅱa
301	60	Male	Smoker	P	T2bN2M0	IIIa
303	60	Male	Smoker	P	T2aN2M0	IIIa
310	51	Male	Smoker	P	T4N0M0	IIIa
313	57	Male	Smoker	P	T2aN1M0	Ⅱa
315	52	Male	Smoker	M	T2aN1M0	Ⅱa
321	62	Male	Smoker	M	T3N1M0	IIIa
341	55	Male	Smoker	P	T2bN2M0	IIIa
343	65	Female	Non-smoker	P	T2aN2M0	IIIa
348	64	Male	Smoker	M	T3N0M0	Ⅱb
361	55	Male	Smoker	M	T2aN0M0	Ⅰb
367	54	Male	Smoker	P	T1bN1M0	Ⅱa
371	58	Male	Smoker	P	T2aN1M0	Ⅱa

375	70	Male	Smoker	W	T2aN0M0	Ⅰb
378	60	Male	Smoker	P	T3N1M0	Ⅲa
387	48	Female	Non-smoker	P	T2bN2M0	Ⅲa
394	54	Male	Smoker	P	T1aN0M0	Ⅰa
400	62	Male	Smoker	P	T1aN0M0	Ⅰa
408	45	Male	Smoker	P	T2aN1M0	Ⅱa
416	56	Female	Non-smoker	P	T2aN2M0	Ⅲa
417	68	Male	Smoker	P	T1bN0M0	Ⅰa
428	57	Male	Smoker	M	T2aN0M0	Ⅰb
431	60	Male	Smoker	M	T2aN0M0	Ⅰb
433	51	Male	Smoker	M	T2aN0M0	Ⅰb
438	70	Male	Smoker	P	T2bN2M0	Ⅲa
451	63	Male	Smoker	M	T1bN0M0	Ⅰa
467	68	Male	Non-smoker	P	T3N0M0	Ⅱb
474	67	Male	Smoker	P	T2aN2M0	Ⅲa
482	61	Male	Smoker	P	T1bN0M0	Ⅰa
503	57	Male	Smoker	M	T2aN0M0	Ⅰb
504	69	Male	Smoker	M	T2aN0M0	Ⅰb
520	63	Male	Smoker	P	T1aN2M0	Ⅲa
523	51	Male	Smoker	P	T2bN2M0	Ⅲa
527	42	Female	Non-smoker	P	T2aN2M0	Ⅲa
534	75	Male	Non-smoker	M	T2bN2M0	Ⅲa
537	70	Male	Smoker	W	T3N1M0	Ⅲa
538	73	Male	Smoker	W	T2bN2M0	Ⅲa
545	59	Male	Smoker	P	T2aN2M0	Ⅲa
553	59	Male	Smoker	P	T2aN0M0	Ⅰb
554	39	Male	Smoker	M	T2aN0M0	Ⅰb
601	49	Male	Smoker	P	T2bN2M0	Ⅲa
611	63	Male	Non-smoker	M	T3N1M0	Ⅲa
623	50	Male	Smoker	P	T2aN0M0	Ⅰb
699	64	Male	Smoker	P	T2aN0M0	Ⅰb
700	62	Male	Smoker	M	T1aN1M0	Ⅱa
715	58	Male	Smoker	P	T2bN0M0	Ⅱa
755	62	Male	Smoker	P	T2aN0M0	Ⅰb
757	55	Male	Smoker	P	T2aN1M0	Ⅱa
804	59	Male	Smoker	P	T2aN0M0	Ⅰb
814	67	Male	Smoker	M	T2aN2M0	Ⅲa
821	47	Male	Smoker	M	T1bN2M0	Ⅲa
830	54	Male	Smoker	M	T2aN2M0	Ⅲa
856	72	Male	Non-smoker	P	T1aN0M0	Ⅰa
863	52	Male	Non-smoker	M	T1aN0M0	Ⅰa
869	61	Female	Smoker	P	T3N2M0	Ⅲa
873	63	Male	Smoker	M	T2bN2M0	Ⅲa
882	69	Male	Smoker	P	T1bN0M0	Ⅰa
886	51	Female	Non-smoker	P	T2aN2M0	Ⅲa
888	69	Male	Smoker	P	T2bN0M0	Ⅱa
896	69	Male	Smoker	P	T1aN0M0	Ⅰa
901	61	Male	Smoker	M	T3N0M0	Ⅱb
909	64	Female	Non-smoker	P	T2aN2M0	Ⅲa
916	55	Male	Smoker	M	T2aN2M0	Ⅲa
935	56	Male	Smoker	M	T2bN2M0	Ⅲa

*The TNM stage and stage grouping are according to UICC 2009 NSCLC staging system. W, well differentiated; M, moderatly differentiated; P, poorly differentiated.

Supplementary Table 2. Clinical information of additional Lung SQCCs for TCS validation

Sample ID	Age (years)	Gender	Smoking status	Differentiation	TNM*	Stage*
1019	83	Female	Non-smoker	P	T2aN0M0	Ⅰb
1021	55	Male	Smoker	M	T2aN0M0	Ⅰb
1038	74	Male	Smoker	P	T2aN1M0	Ⅱa
1043	48	Male	Smoker	P	T1bN0M0	Ⅰa
1080	59	Female	Non-smoker	P	T3N0M0	Ⅱb
1088	67	Male	Smoker	P	T2aN0M0	Ⅰb
1096	52	Male	Smoker	M	T3N0M0	Ⅱb
1108	76	Male	Smoker	M	T2bN0M0	Ⅱa
1115	67	Male	Smoker	M	T2aN1M0	Ⅱa
1125	60	Male	Smoker	M	T2aN2M0	Ⅲa
1149	56	Male	Smoker	W	T2aN0M0	Ⅰb
1167	48	Male	Smoker	M	T2aN1M0	Ⅱa
1175	64	Male	Smoker	P	T3N0M0	Ⅱb
1185	58	Male	Smoker	P	T2aN2M0	Ⅲa
1194	58	Male	Smoker	P	T2aN0M0	Ⅰb
1217	70	Male	Smoker	M	T1aN0M0	Ⅰa
1223	58	Male	Smoker	P	T1bN2M0	Ⅲa
1233	56	Male	Smoker	M	T1bN2M0	Ⅲa
1239	67	Male	Smoker	W	T3N2M0	Ⅲa
1241	59	Male	Smoker	W	T4N0M0	Ⅲa
1246	75	Female	Smoker	M	T2bN2M0	Ⅲa
1250	59	Male	Smoker	P	T2aN1M0	Ⅱa
1251	60	Male	Non-smoker	M	T2bN1M0	Ⅱb
1252	57	Male	Smoker	P	T2aN0M0	Ⅰb
1257	54	Male	Smoker	P	T2aN1M0	Ⅱa
1264	71	Male	Non-smoker	M	T2aN1M0	Ⅱa
1270	56	Male	Smoker	P	T3N2M0	Ⅲa
1276	72	Male	Smoker	M	T2bN0M0	Ⅱa
1285	79	Male	Smoker	W	T1aN0M0	Ⅰa
1292	71	Male	Smoker	P	T1bN0M0	Ⅰa
1294	58	Male	Smoker	M	T2bN0M0	Ⅱa
1296	70	Male	Smoker	M	T2bN0M0	Ⅱa
1297	51	Male	Smoker	M	T2aN1M0	Ⅱa
1300	62	Male	Smoker	P	T2aN0M0	Ⅰb
1314	63	Male	Smoker	P	T3N0M0	Ⅱb
1322	62	Male	Smoker	P	T2bN1M0	Ⅱb
1323	63	Male	Smoker	P	T2aN0M0	Ⅰb
1325	51	Male	Smoker	M	T2aN1M0	Ⅱa
1338	78	Male	Smoker	M	T3N0M0	Ⅱb
1344	69	Male	Non-smoker	P	T3N2M0	Ⅲa
1347	57	Male	Smoker	P	T2aN2M0	Ⅲa
1348	60	Male	Smoker	M	T1aN1M0	Ⅱa
1351	65	Male	Smoker	P	T2aN2M0	Ⅲa
1355	60	Male	Smoker	P	T3N2M0	Ⅲa
1361	64	Male	Smoker	P	T3N0M0	Ⅱb
1368	59	Male	Smoker	P	T2aN2M0	Ⅲa
1369	63	Male	Smoker	P	T2bN0M0	Ⅱa
1370	68	Male	Smoker	W	T2bN0M0	Ⅱa
1378	67	Male	Smoker	M	T2aN0M0	Ⅰb

1382	60	Male	Smoker	W	T1bN0M0	Ⅰa
1387	62	Male	Smoker	M	T3N1M0	ⅢA
1389	70	Male	Smoker	M	T2aN1M0	Ⅱa
1395	57	Male	Smoker	M	T2aN1M0	Ⅱa
1396	79	Male	Smoker	P	T2aN0M0	Ⅰb
1405	63	Female	Non-smoker	P	T3N1M0	Ⅲa
1433	66	Male	Smoker	M	T2aN0M0	Ⅰb
1436	51	Male	Smoker	P	T1aN0M0	Ⅰa
1447	54	Male	Smoker	M	T2aN0M0	Ⅰb
1461	64	Female	Non-smoker	M	T2aN2M0	Ⅲa
1477	45	Female	Non-smoker	P	T3N2M0	Ⅲa
1485	56	Male	Smoker	P	T2aN2M0	Ⅲa
1488	49	Female	Non-smoker	P	T1bN2M0	Ⅲa
1489	49	Male	Smoker	P	T3N2M0	Ⅲa
1507	67	Male	Smoker	M	T3N0M0	Ⅱb
1530	76	Male	Smoker	M	T2aN0M0	Ⅰb
1535	70	Male	Smoker	P	T1bN0M0	Ⅰa
1540	87	Male	Smoker	P	T1bN0M0	Ⅰa
1556	45	Male	Smoker	P	T2aN2M0	Ⅲa
1557	58	Male	Smoker	P	T2aN1M0	Ⅱa
1567	71	Male	Smoker	M	T1bN0M0	Ⅰa
1570	54	Male	Smoker	M	T1bN1M0	Ⅱa
1587	56	Male	Smoker	M	T2aN2M0	Ⅲa
1589	54	Male	Smoker	P	T2bN1M0	Ⅱb
1598	53	Male	Smoker	M	T2bN0M0	Ⅱa
1630	53	Male	Smoker	M	T2aN2M0	Ⅲa
1660	44	Male	Smoker	M	T1bN0M0	Ⅰa
1673	56	Male	Smoker	P	T1bN1M0	Ⅱa
1678	62	Male	Smoker	P	T2aN0M0	Ⅰb
1679	60	Male	Smoker	P	T2aN1M0	Ⅱa
1681	59	Male	Smoker	P	T2aN1M0	Ⅱa
1689	60	Male	Smoker	P	T1bN0M0	Ⅰa
1691	72	Male	Smoker	M	T2bN0M0	Ⅱa
1723	57	Male	Smoker	M	T2bN2M0	Ⅲa
1730	56	Male	Smoker	P	T2aN0M0	Ⅰb
1740	56	Male	Smoker	M	T2bN1M0	Ⅱb
1745	60	Male	Smoker	P	T2aN0M0	Ⅰb
1754	43	Male	Smoker	M	T1aN0M0	Ⅰa
1762	46	Male	Smoker	M	T1bN0M0	Ⅰa
1763	62	Male	Smoker	M	T3N0M0	Ⅱb
1780	62	Male	Smoker	M	T2aN0M0	Ⅰb
1785	74	Male	Smoker	M	T2bN0M0	Ⅱa
1796	71	Male	Smoker	P	T2aN0M0	Ⅰb
1797	74	Male	Smoker	M	T2bN2M0	Ⅲa
1813	51	Male	Smoker	P	T2aN2M0	Ⅲa
1837	55	Female	Non-smoker	P	T2aN0M0	Ⅰb
1848	67	Male	Smoker	P	T3N0M0	Ⅱb
1855	62	Male	Smoker	M	T3N1M0	Ⅲa
1862	63	Male	Smoker	P	T1aN0M0	Ⅰa

TCS, Target-Capture Sequencing. *The TNM stage and stage grouping are according to UICC 2009 NSCLC staging system. W, well differentiated; M, moderately differentiated; P, poorly
